# Tebuconazol und Penconazol – Bestimmung von TEB-OH, TEB-COOH, PEN-OH und PEN-COOH in Urin mittels LC-MS/MS: Biomonitoring-Methode

**DOI:** 10.34865/bi10753496d11_1or

**Published:** 2026-03-30

**Authors:** Elisa Polledri, Rosa Mercadante, Silvia Fustinoni, Laura Kuhlmann, Thomas Göen, Andrea Hartwig, MAK Commission

**Affiliations:** 1 Laboratory of Environmental and Occupational Toxicology. Department of Clinical Sciences and Community Health. University of Milano and Fondazione IRCCS Ca Granda Ospedale Maggiore Policlinico Via Francesco Sforza 35 20122 Mailand Italien; 2 Friedrich-Alexander-Universität Erlangen-Nürnberg. Institut und Poliklinik für Arbeits-, Sozial- und Umweltmedizin Henkestraße 9–11 91054 Erlangen Deutschland; 3 Institut für Angewandte Biowissenschaften. Abteilung Lebensmittelchemie und Toxikologie. Karlsruher Institut für Technologie (KIT) Adenauerring 20a, Geb. 50.41 76131 Karlsruhe Deutschland; 4 Ständige Senatskommission zur Prüfung gesundheitsschädlicher Arbeitsstoffe. Deutsche Forschungsgemeinschaft, Kennedyallee 40, 53175 Bonn, Deutschland. Weitere Informationen: Ständige Senatskommission zur Prüfung gesundheitsschädlicher Arbeitsstoffe | DFG

**Keywords:** Tebuconazol, Penconazol, Biomonitoring, Urin, LC-MS/MS

## Abstract

The working group “Analyses in Biological Materials” of the German Senate Commission for the Investigation of Health Hazards of Chemical Compounds in the Work Area (MAK Commission) developed and verified this biomonitoring method for the measurement of the most important urinary metabolites of the triazole fungicides tebuconazole and penconazole. Specifically, this method determines (*RS*)-5-(4-chlorophenyl)-2,2-dimethyl-3-(1*H*-1,2,4-triazol-1-ylmethyl)-1,3-pentanediol (TEB-OH) and (*RS*)-5-(4-chlorophenyl)-2,2-dimethyl-3-(1*H*-1,2,4-triazol-1-ylmethyl)-3-olpentanoic acid (TEB-COOH) as well as 4-(2,4-dichlorophenyl)-5-[1,2,4]-triazol-1-ylpentanol (PEN-OH) and 4-(2,4-dichlorophenyl)-5-[1,2,4]-triazol-1-ylpentanoic acid (PEN-COOH) in urine. After adding isotope-labelled internal standards, the samples are enzymatically hydrolysed to release the analytes from the glucuronide and sulfate conjugates. After online purification, the analytes are separated by liquid chromatography and analysed using tandem mass spectrometry. Calibration is performed using calibration standards prepared in pooled urine and processed analogously to the samples to be analysed. The method provides reliable and accurate analytical results, as shown by the good precision data with standard deviations in the range of 0.1–10.7%. Good accuracy data were obtained with mean relative recoveries in the range of 100–107%. The method is selective, sensitive, and provides quantitation limits of 0.3 μg/l for TEB-OH and TEB-COOH and of 1.0 μg/l for PEN-OH and PEN-COOH.

## 1 Kenndaten der Methode

**Table:** 

**Matrix**	Urin		
**Analytisches Messprinzip**	Flüssigkeitschromatographie mit Tandem-Massenspektrometrie (LC‑MS/MS)		
**Parameter und entsprechende Arbeitsstoffe**			
**Arbeitsstoff**	**CAS‑Nr.**	**Parameter**	**CAS‑Nr.**
Tebuconazol(1‑(4‑Chlorphenyl)-4,4‑dimethyl-3‑(1*H*‑1,2,4‑triazol-1‑ylmethyl)-pentan-3‑ol)	107534‑96‑3	(*RS*)‑5‑(4‑Chlorphenyl)-2,2‑dimethyl-3‑(1*H*‑1,2,4‑triazol-1‑ylmethyl)-1,3‑pentandiol (TEB‑OH)	212267‑64‑6
		(*RS*)-5‑(4‑Chlorphenyl)-2,2‑dimethyl-3‑(1*H*‑1,2,4‑triazol-1‑ylmethyl)-3‑olpentansäure (TEB‑COOH)	1631977-20-2
Penconazol((*RS*)-1‑[2‑(2,4‑Dichlorphenyl)-pentyl]-1*H*‑1,2,4‑triazol)	66246‑88‑6	4‑(2,4‑Dichlorphenyl)-5‑[1,2,4]-triazol-1‑ylpentanol (PEN‑OH)	1945983-65-2
		4‑(2,4‑Dichlorphenyl)-5‑[1,2,4]‑triazol-1‑ylpentansäure (PEN‑COOH)	1945983-66-3

### Zuverlässigkeitskriterien

#### TEB‑OH

**Table:** 

Präzision in der Serie:	Standardabweichung (rel.)	*s_w_* = 0,1–1,7 % bzw. 0,1–1,4 %
	Streubereich	*u* = 0,28–4,72 % bzw. 0,28–3,89 %
	bei einer dotierten Konzentration von 20 μg oder 150 μg TEB‑OH pro Liter Urin und n = 5 Bestimmungen	
Präzision von Tag zu Tag:	Standardabweichung (rel.)	*s_w_* = 1,0 % bzw. 0,8 %
	Streubereich	*u* = 2,45 % bzw. 1,96 %
	bei einer dotierten Konzentration von 20 μg oder 150 μg TEB‑OH pro Liter Urin und n = 7 Bestimmungen	
Richtigkeit:	Wiederfindung (rel.)	*r* = 100 % bzw. 100 %
	bei einer dotierten Konzentration von 20 μg oder 150 μg TEB‑OH pro Liter Urin und n = 5 Bestimmungen	
Nachweisgrenze:	0,2 μg TEB‑OH pro Liter Urin	
Bestimmungsgrenze:	0,3 μg TEB‑OH pro Liter Urin	

#### TEB‑COOH

**Table:** 

Präzision in der Serie:	Standardabweichung (rel.)	*s_w_* = 0,4–3,1 % bzw. 0,1–0,9 %
	Streubereich	*u* = 1,11–8,61 % bzw. 0,28–2,50 %
	bei einer dotierten Konzentration von 8 μg oder 60 μg TEB‑COOH pro Liter Urin und n = 5 Bestimmungen	
Präzision von Tag zu Tag:	Standardabweichung (rel.)	*s_w_* = 1,6 % bzw. 1,4 %
	Streubereich	*u* = 3,92 % bzw. 3,43 %
	bei einer dotierten Konzentration von 8 μg oder 60 μg TEB‑COOH pro Liter Urin und n = 7 Bestimmungen	
Richtigkeit:	Wiederfindung (rel.)	*r* = 100 % bzw. 101 %
	bei einer dotierten Konzentration von 8 μg oder 60 μg TEB‑COOH pro Liter Urin und n = 5 Bestimmungen	
Nachweisgrenze:	0,1 μg TEB‑COOH pro Liter Urin	
Bestimmungsgrenze:	0,3 μg TEB‑COOH pro Liter Urin	

#### PEN‑OH

**Table:** 

Präzision in der Serie:	Standardabweichung (rel.)	*s_w_* = 3,3–4,2 % bzw. 2,1–3,5 %
	Streubereich	*u* = 9,16–11,7 % bzw. 5,83–9,72 %
	bei einer dotierten Konzentration von 5 μg oder 150 μg PEN‑OH pro Liter Urin und n = 5 Bestimmungen	
Präzision von Tag zu Tag:	Standardabweichung (rel.)	*s_w_* = 10,7 % bzw. 4,1 %
	Streubereich	*u* = 26,2 % bzw. 10,0 %
	bei einer dotierten Konzentration von 5 μg oder 150 μg PEN‑OH pro Liter Urin und n = 7 Bestimmungen	
Richtigkeit:	Wiederfindung (rel.)	*r* = 100 % bzw. 105 %
	bei einer dotierten Konzentration von 5 μg oder 150 μg PEN‑OH pro Liter Urin und n = 5 Bestimmungen	
Nachweisgrenze:	0,5 μg PEN‑OH pro Liter Urin	
Bestimmungsgrenze:	1,0 μg PEN‑OH pro Liter Urin	

#### PEN‑COOH

**Table:** 

Präzision in der Serie:	Standardabweichung (rel.)	*s_w_* = 3,4–7,5 % bzw. 2,1–3,4 %
	Streubereich	*u* = 9,44–20,8 % bzw. 5,83–9,44 %
	bei einer dotierten Konzentration von 5 μg oder 150 μg PEN‑COOH pro Liter Urin und n = 5 Bestimmungen	
Präzision von Tag zu Tag:	Standardabweichung (rel.)	*s_w_* = 8,7 % bzw. 7,4 %
	Streubereich	*u* = 21,3 % bzw. 18,1 %
	bei einer dotierten Konzentration von 5 μg oder 150 μg PEN‑COOH pro Liter Urin und n = 7 Bestimmungen	
Richtigkeit:	Wiederfindung (rel.)	*r* = 106 % bzw. 107 %
	bei einer dotierten Konzentration von 5 μg oder 150 μg PEN‑COOH pro Liter Urin und n = 5 Bestimmungen	
Nachweisgrenze:	0,5 μg PEN‑COOH pro Liter Urin	
Bestimmungsgrenze:	1,0 μg PEN‑COOH pro Liter Urin	

## 2 Allgemeine Informationen zu Tebuconazol und Penconazol

Tebuconazol und Penconazol sind Triazolfungizide, die die Sterolbiosynthese in Pilzen hemmen (Demethylierungshemmer). Um den echten Mehltau zu bekämpfen, werden sie beim Anbau verschiedener Kulturpflanzen eingesetzt, insbesondere im Gemüse- und Obstbau einschließlich dem Weinbau. Tebuconazol und Penconazol sind in vielen auf dem Markt erhältlichen Fungizidprodukten als Wirkstoff enthalten, wobei Tebuconazol unter den zehn wichtigsten Fungizidwirkstoffen im Pflanzenschutz aufgeführt ist (Eurostat 2007).

Das *Joint Meeting on Pesticide Residues *(WHO/FAO) hatte 1994 Triazole als für den Menschen unbedenklich eingestuft (WHO und FAO 1994). Später gab es jedoch Hinweise darauf, dass diese Substanzen in hohen Dosen Fehlbildungen bei Tieren verursachen können (Giavini und Menegola 2010). Für Tebuconazol schlug die Europäische Behörde für Lebensmittelsicherheit (*European Food Safety Authority*, EFSA) 2008 folgende Einstufung vor: „Kann das Kind im Mutterleib möglicherweise schädigen“ (EFSA 2008). Tebuconazol und Penconazol wurden von der MAK-Kommission bisher noch nicht bewertet.

In der Landwirtschaft sind die Arbeiter während der Ausbringung entsprechender Fungizidprodukte gegen Tebuconazol und Penconazol exponiert, aber auch Anwohner in der Nähe behandelter Flächen können betroffen sein. Darüber hinaus kann die Allgemeinbevölkerung durch den Verzehr von belasteten Lebensmitteln exponiert sein (NCBI 2026). Die vorliegenden Daten weisen darauf hin, dass Penconazol und Tebuconazol nicht nur inhalativ sondern auch oral und dermal gut resorbiert werden (Mercadante et al. 2019; Oerlemans et al. 2019; Sams et al. 2016).

**Abb. 1:**
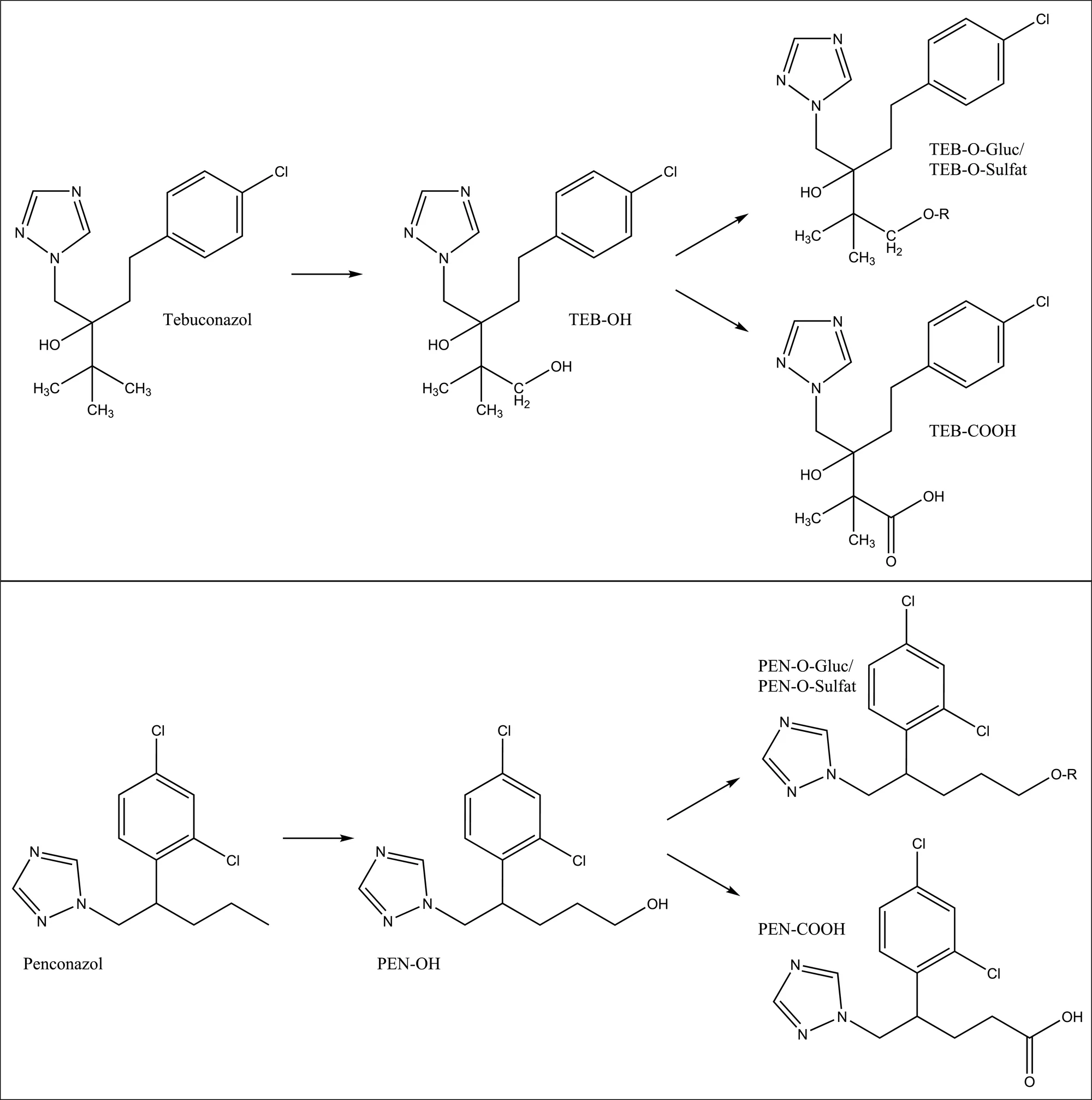
Strukturformeln der Fungizide Tebuconazol und Penconazol sowie ihrer Hauptmetaboliten

Oral oder dermal aufgenommenes **Tebuconazol** wird im menschlichen Körper zunächst zu (*RS*)-5‑(4‑Chlorphenyl)-2,2‑dimethyl-3‑(1*H*‑1,2,4‑triazol-1‑ylmethyl)-1,3‑pentandiol (TEB‑OH) oxidiert und dieses anschließend glucuronidiert, sulfatiert oder weiter zu (*RS*)-5‑(4‑Chlorphenyl)-2,2‑dimethyl-3‑(1*H*‑1,2,4‑triazol-1‑ylmethyl)-3‑olpentansäure (TEB‑COOH) oxidiert (WHO und FAO 1994). TEB-OH-Konjugate sowie freies TEB‑OH und TEB‑COOH stellen die Hauptmetaboliten des Tebuconazols in Humanurin dar (Abbildung 1). Daten zum Metabolismus und zur Toxikokinetik von Tebuconazol beim Menschen wurden erstmals von Oerlemans et al. (2019) publiziert. Nach kontrollierter oraler und dermaler Verabreichung von 1,5 mg bzw. 2,5 mg Tebuconazol wurde eine maximale Ausscheidung von TEB‑OH im Urin nach 1,4 h und 20,8 h sowie eine Eliminationshalbwertszeit von 7,8 h bzw. 15,8 h gefunden. In 48 h wurden 38 % bzw. 1 % der applizierten Menge als TEB‑OH ausgeschiedenen.

In einer Feldstudie wurden Urinproben von sieben beruflich gegen Tebuconazol exponierten Arbeitern gewonnen und auf ihre Gehalte an TEB‑OH und TEB‑COOH analysiert. In fünf Urinproben lagen die TEB‑OH-Gehalte ohne Hydrolyse oberhalb der Bestimmungsgrenze. Nach Hydrolyse waren die TEB-OH-Gehalte in vier der fünf Proben 6‑ bis 77‑fach höher als in den nicht hydrolysierten Proben. In der fünften Probe war der TEB‑OH-Gehalt nach Hydrolyse fast unverändert. Auch die Konzentration des freien TEB‑COOH lag nach Hydrolyse höher, wobei der Effekt nicht so ausgeprägt war wie beim TEB‑OH. Die Ergebnisse zeigen, dass der Anteil der Konjugate interindividuell stark variieren kann. Die TEB‑OH-Konzentrationen waren in den hydrolysierten Proben mit 9,8 bis 473 μg/l etwa vierfach so hoch wie die TEB‑COOH-Konzentrationen von 2,7 bis 159 μg/l (Mercadante et al. 2014).

**Penconazol** wird, einmal in den menschlichen Körper aufgenommen, ebenfalls oxidiert, wobei zunächst 4‑(2,4‑Dichlorphenyl-5‑[1,2,4]‑triazol-1‑ylpentanol (PEN‑OH) entsteht. Dieser Metabolit wird zum entsprechenden Glucuronid oder Sulfat konjugiert oder weiter zur Säure (4‑(2,4‑Dichlorphenyl)-5‑[1,2,4]‑triazol-1‑ylpentansäure; PEN‑COOH) oxidiert (WHO und FAO 1993). PEN‑OH‑Konjugate, sowie freies PEN‑OH und PEN‑COOH stellen die wichtigsten Penconazolmetaboliten im menschlichen Urin dar (Abbildung 1). In einer Studie von Sams et al. (2016) wurde die Verstoffwechselung von Penconazol nach kontrollierter oraler Verabreichung von 0,03 mg Penconazol/kg Körpergewicht an drei Probanden untersucht. Die maximalen Ausscheidungsraten der beiden Metaboliten PEN‑OH und PEN‑COOH in Urin wurden innerhalb der ersten 2 Stunden nach Applikation erreicht. Die mittleren Eliminationshalbwertszeiten von PEN‑OH und PEN‑COOH betrugen dabei 3,1 h bzw. 3,7 h. In 48 h wurden im Mittel 47 % der applizierten Dosis ausgeschieden, wobei davon 83 % auf PEN‑OH und 17 % auf PEN‑COOH entfielen.

Da in einer Studie an Arbeitern, die Penconazol in Weinbaugebieten ausbrachten, die innere Belastung gut mit der aktuellen dermalen Exposition korrelierte, kann für Penconazol eine zu Tebuconzol ähnliche Aufnahme und Toxikokinetik nach dermaler Exposition angenommen werden (Mercadante et al. 2019).

Um die berufliche Exposition gegen Penconazol abzuschätzen, wurden Urinproben von 22 beruflich exponierten Arbeitern mit der vorliegenden Methode analysiert. Die Konzentrationen der Penconazolmetaboliten in den individuellen Urinen waren sehr unterschiedlich, wobei sich diese Variabilität durch Hydrolyse der Proben deutlich verringerte (Mercadante et al. 2016). Die Beschäftigten wurden nach Penconazolausbringung sowie nach Wiedereintritt in die mit Penconazol behandelten Bereiche untersucht. An bis zu vier aufeinanderfolgenden Arbeitstagen wurde das Spritzmittel angesetzt und ausgebracht (n = 42) und die behandelten Bereiche wurden zwölfmal wieder betreten. Urinproben wurden vor der ersten Ausbringung, während des Ansetzens und während der Ausbringung und bis zu 48 h nach der letzten Schicht gesammelt. Die PEN‑OH-Konzentrationen lagen deutlich über den PEN‑COOH-Konzentrationen und umfassten 24 h nach Ausbringung einen Bereich von 15,6 bis 27,6 μg PEN‑OH/l sowie 2,5 bis 10,2 μg PEN-COOH/l (Mercadante et al. 2019).

In Tabelle 1 sind exemplarisch Hintergrundkonzentrationen von Tebuconazol und TEB‑OH im Urin der Allgemeinbevölkerung aufgeführt. Tabelle 2 zeigt beispielhaft Konzentrationen von Tebuconazol, TEB‑OH und TEB‑COOH im Urin beruflich exponierter Personen. In Tabelle 3 sind exemplarisch Hintergrundkonzentrationen von PEN‑COOH im Urin der Allgemeinbevölkerung und in Tabelle 4 beispielhafte Konzentrationen von PEN‑OH und PEN‑COOH im Urin beruflich exponierter Personen angegeben.

**Tab.1:** Tebuconazol und seine Metaboliten im Urin der nicht beruflich exponierten Allgemeinbevölkerung

Studienkollektiv (Land; n; Probe)	Analyten	DF [%]	QF [%]	NWG [μg/l]	BG [μg/l]	GM [μg/l]	Median [μg/l]	P95 [μg/l]	Bereich [μg/l]	Analysemethode	Literatur
Schwangere Frauen (Argentinien; 89; erster Morgenurin)	TEB	12,4	8,9	0,3	1,0	n. a.	n. a.	n. a.	0,1–0,3	GC-MS/MS	Racca et al. 2025
Kinder im Alter von 7–9 Jahren (Polen; 399; Spontanurin)	TEB	12	n. a.	0,041	n. a.	< BG	< BG	0,056^a)^	< BG–0,076^a)^	UPLC-MS/MS	Bustamante et al. 2025
	TEB-OH	96	n. a.	0,064	n. a.	0,3^a)^	0,35^a)^	1,8^a)^	< BG–19^a)^		
Schwangere Frauen (Deutschland; 587; erster Morgenurin)	TEB	1	n. a.	n. a.	n. a.	n. a.	0,3	n. a.	n. a.	HPLC-HRMS	Huber et al. 2024
	TEB-(OH)_2_	57	n. a.	n. a.	n. a.	n. a.	n. a.	n. a.	n. a.		
Erwachsene (EU; 110; erster Morgenurin)	TEB-OH	n. a.	98,2	0,02	0,05	n. a.	0,47	1,74	n. a.	HPLC-MS/MS	Šulc et al. 2022
Schulkinder (EU; 110; erster Morgenurin)	TEB-OH	n. a.	99,1	0,02	0,05	n. a.	0,44	1,77	n. a.		

BG: Bestimmungsgrenze; DF: Nachweishäufigkeit (*detection frequency*, gemessene Werte oberhalb der NWG); GM: geometrischer Mittelwert; n: Probenanzahl; n. a.: nicht angegeben; NWG: Nachweisgrenze; P95: 95. Perzentil; QF: Bestimmungshäufigkeit (*quantitation frequency*, gemessene Werte oberhalb der BG); TEB: Tebuconazol

^a)^ unter Verwendung von BG/2 für Werte unterhalb der BG berechnet

**Tab.2:** Tebuconazol und seine Metaboliten im Urin exponierter Arbeiter

Studienkollektiv (Land; n; Probe)	Analyten	DF [%]	QF [%]	NWG [μg/l]	BG [μg/l]	GM (GSD) [μg/l]	Median [μg/l]	P95 [μg/l]	Bereich [μg/l]	Analyse⁠methode	Literatur
Landarbeiter (Mexico; 105; Spontanurin)	TEB-OH	76	n. a.	0,10	n. a.	0,36	0,34	n. a.	< NWG–4,17	LC-MS/MS	Alcalá et al. 2024
Weinbauarbeiter (Italien; 3; Spontanurin 24 h nach 2. Arbeitsschicht)	TEB	n. a.	n. a.	0,5	1,5	n. a.	4,6	n. a.	3,7–6,0	LC-MS/MS	Fustinoni et al. 2014
	TEB-OH	n. a.	n. a.	0,2	0,3	n. a.	249,0	n. a.	58,3–383,6		
	TEB-COOH	n. a.	n. a.	0,1	0,3	n. a.	51,1	n. a.	17,3–100,4		
Weinbauarbeiter (Italien; 7; Spontanurin 24 h nach Ausbringung)	TEB-OH	n. a.	n. a.	0,2	0,3	125,6±175,3^a)^	n. a.	n. a.	n. a.	LC-MS/MS	Mercadante et al. 2014
	TEB-COOH	n. a.	n. a.	0,1	0,3	34,5±56,7^a)^	n. a.	n. a.	n. a.		
Landarbeiter (Costa Rica; 299; Spontanurin)	TEB-OH	95,6–97,1	n. a.	0,05	n. a.	0,91 (3,44)	0,88	n. a.	< NWG–35,54	LC-MS/MS	Rodríguez-Zamora et al. 2024

BG: Bestimmungsgrenze; DF: Nachweishäufigkeit (*detection frequency*, gemessene Werte oberhalb der NWG); GM: geometrischer Mittelwert; GSD: geometrische Standardabweichung; n: Probenanzahl; n. a.: nicht angegeben; NWG: Nachweisgrenze; P95: 95. Perzentil; QF: Bestimmungshäufigkeit (*quantitation frequency*, gemessene Werte oberhalb der BG); TEB: Tebuconazol

a) arithmetischer Mittelwert ± Standardabweichung

**Tab.3:** PEN-COOH im Urin der nicht beruflich exponierten Allgemeinbevölkerung

Studienkollektiv (Land; n; Probe)	Analyten	DF [%]	QF [%]	NWG [μg/l]	BG [μg/l]	GM [μg/l]	Median [μg/l]	P95 [μg/l]	Bereich [μg/l]	Analysemethode	Literatur
Bewohner ländlicher Gebiete (Vereinigtes Königreich; 483; Spontanurin außerhalb Sprühsaison)	PEN-COOH	12	n. a.	0,25	n. a.	n. a.	n. a.	0,22	< NWG–1,73	LC-MS/MS	Sams et al. 2016
Bewohner ländlicher Gebiete (Vereinigtes Königreich; 556; Spontanurin in der Sprühsaison)	PEN-COOH	10	n. a.	0,25	n. a.	n. a.	n. a.	0,29	< NWG–1,19		

BG: Bestimmungsgrenze; DF: Nachweishäufigkeit (*detection frequency*, gemessene Werte oberhalb der NWG); GM: geometrischer Mittelwert; n: Probenanzahl; n. a.: nicht angegeben; NWG: Nachweisgrenze; P95: 95. Perzentil; PEN: Penconazol; QF: Bestimmungshäufigkeit (*quantitation frequency*, gemessene Werte oberhalb der BG)

**Tab.4:** Penconazol und seine Metaboliten im Urin exponierter Arbeiter

Studienkollektiv (Land; n; Probe)	Analyten	DF [%]	QF [%]	NWG [μg/l]	BG [μg/l]	GM (GSD) [μg/l]	Median [μg/l]	P95 [μg/l]	Bereich [μg/l]	Analysemethode	Literatur
Landarbeiter (Italien; 5; Spontanurin)	PEN	n. a.	n. a.	n. a.	2,0	n. a.	< BG	n. a.	< BG–3,1	LC-MS/MS	Mercadante et al. 2016
	PEN-OH	n. a.	n. a.	n. a.	1,0	n. a.	290,0	n. a.	230,1–464,3		
	PEN-COOH	n. a.	n. a.	n. a.	1,0	n. a.	7,9	n. a.	5,2–16,7		
Weinbauarbeiter (Italien; 22; 24 h-Sammelurin 25–48 h nach der Schicht)	PEN-OH	n. a.	n. a.	n. a.	1,0	n. a.	9,4	n. a.	1,2–237,0	LC-MS/MS	Mercadante et al. 2019
	PEN-COOH	n. a.	n. a.	n. a.	1,0	n. a.	1,8	n. a.	< 1,0–50,9		
Landarbeiter (Costa Rica; 299; Spontanurin)	PEN-OH	38,9–44,0	n. a.	0,05	n. a.	0,06 (2,09)	0,06	n. a.	< NWG–0,63	LC-MS/MS	Rodríguez-Zamora et al. 2024

BG: Bestimmungsgrenze; DF: Nachweishäufigkeit (*detection frequency*, gemessene Werte oberhalb der NWG); GM: geometrischer Mittelwert; GSD: geometrische Standardabweichung; n: Probenanzahl; n. a.: nicht angegeben; NWG: Nachweisgrenze; P95: 95. Perzentil; PEN: Penconazol; QF: Bestimmungshäufigkeit (*quantitation frequency*, gemessene Werte oberhalb der BG)

## 3 Grundlagen der Methode

Die hier beschriebene analytische Methode ermöglicht die selektive und sensitive Bestimmung von TEB‑OH, TEB‑COOH, PEN‑OH und PEN‑COOH in Urin. Nach Zugabe der markierten internen Standards (Tebuconazol‑D_6_ und Penconazol‑D_7_) werden die Proben enzymatisch hydrolysiert, um die Glucuronid- und Sulfatkonjugate zu spalten. Nach Online-Aufreinigung werden die Analyten mittels Flüssigkeitschromatographie getrennt und mittels Tandem-Massenspektrometrie quantifiziert. Die Kalibrierung erfolgt mit Kalibrierstandards, die in Poolurin angesetzt und in der gleichen Weise aufgearbeitet werden, wie die zu analysierenden Proben.

## 4 Geräte, Chemikalien und Lösungen

### 4.1 Geräte

- LC‑Anlage bestehend aus einem TurboFlow-System mit zwei Gradientenpumpen, einem Eluenten-Entgaser und einem Autosampler (z. B. Thermo Fisher Scientific S.p.A., Rodano, Italien)
- Tandem-Massenspektrometer (z. B. TSQ Quantum Access, Thermo Fisher Scientific S.p.A., Rodano, Italien)
- TurboFlow^TM^-Säule (z. B. Nr. CH-953288, TurboFlow^TM^-Cyclone-Säule, Länge: 50 mm, Innendurchmesser: 0,5 mm, Thermo Fisher Scientific S.p.A., Rodano, Italien)
- Analytische LC‑Säule (z. B. Nr. 25403-052130, Hypersil Gold PFP, Länge: 50 mm, Innendurchmesser: 2,1 mm, Partikelgröße: 3 μm, Thermo Fisher Scientific  S.p.A., Rodano, Italien)
- Laborschüttler (z. B. VIBROMIX, Domel Holding, d.o.o., Železniki, Slowenien)
- Analysenwaage (z. B. Mettler-Toledo GmbH, Gießen)
- Trockenschrank (z. B. Modell STZ 5.4, Ettore Pasquali S.r.l., Mailand, Italien)
- Verschiedene Messkolben, Bechergläser und Messzylinder (z. B. Schott AG, Mainz)
- Verschiedene Pipetten und Multipetten^®^ mit passenden Pipettenspitzen (z. B. Gilson International BV Deutschland, Berlin oder Eppendorf AG, Hamburg)
- 2‑ml‑Bördelrandgläschen mit Bördelkappen (z. B. Agilent Technologies Germany GmbH & Co. KG, Waldbronn)
- Urinbecher (z. B. SARSTEDT AG & Co. KG, Nümbrecht)

### 4.2 Chemikalien

Wenn nicht anders angegeben, sind alle genannten Chemikalien mindestens in p. a.-Qualität zu verwenden.

- Aceton, EMSURE^®^ (z. B. Nr. 1.00014, Merck KGaA, Darmstadt)
- Acetonitril, SupraSolv^®^ (z. B. Nr. 100017, Merck KGaA, Darmstadt)
- Ammoniumhydroxid-Lösung, 28–30 % NH_3_ (z. B. Nr. 221228, Merck KGaA, Darmstadt)
- Essigsäure (100 %, Eisessig) (z. B. Nr. 1.00063, Merck KGaA, Darmstadt)
- *β*‑Glucuronidase aus *Helix pomatia*, Typ H‑2 (≥ 100 000 U/ml Glucuronidaseaktivität sowie ≤ 7500 U/ml Sulfataseaktivität) (z. B. Nr. G7017, Merck KGaA, Darmstadt)
- Methanol, LiChrosolv^®^ (z. B. Nr. 1.06007, Merck KGaA, Darmstadt)
- Natriumacetat‑Trihydrat, EMSURE^®^ (z. B. Nr. 1.06267, Merck KGaA, Darmstadt)
- 2‑Propanol, LiChrosolv^®^ (z. B. Nr. 1.02781, Merck KGaA, Darmstadt)
- Hochreines Wasser (z. B. Milli‑Q^®^ Reinstwassersystem, Merck KGaA, Darmstadt)
- Tebuconazol‑D_6_, 100 μg/ml in Aceton (z. B. Nr. DRE‑XA17178710AC, Dr. Ehrenstorfer, LGC Standards GmbH, Wesel)
- (*RS*)-5‑(4‑Chlorphenyl)-2,2‑dimethyl-3‑(1*H*‑1,2,4‑triazol-1‑ylmethyl)-1,3‑pentandiol (TEB‑OH), technische Qualität, 97,5 % (freundlicherweise zur Verfügung gestellt von Bayer CropScience Deutschland GmbH, Monheim)
- (*RS*)-5‑(4‑Chlorphenyl)-2,2‑dimethyl-3‑(1*H*‑1,2,4‑triazol-1‑ylmethyl)-3‑olpentansäure (TEB‑COOH), technische Qualität, 97,5 % (freundlicherweise zur Verfügung gestellt von Bayer CropScience Deutschland GmbH, Monheim)
- Penconazol‑D_7_ (z. B. Nr. AABH9A22149F, Merck KGaA, Darmstadt)
- 4‑(2,4‑Dichlorphenyl)-5‑[1,2,4]‑triazol-1‑ylpentanol (PEN‑OH), technische Qualität, 98 % (freundlicherweise zur Verfügung gestellt von Syngenta Group, Cambridge, Vereinigtes Königreich)
- 4‑(2,4‑Dichlorphenyl)-5‑[1,2,4]-triazol-1‑ylpentansäure (PEN‑COOH), technische Qualität, 98 % (freundlicherweise zur Verfügung gestellt von Syngenta Group, Cambridge, Vereinigtes Königreich)

### 4.3 Lösungen

- Eluent C (Acetonitril : 2-Propanol : Aceton (45 : 45 : 10; V/V/V))
  In einen 500‑ml-Messkolben werden 225 ml Acetonitril, 225 ml 2-Propanol sowie 50 ml Aceton gegeben und durch Umschütteln gemischt.
- Eluent D (Acetonitril : Ammoniumhydroxid (99,97 : 0,03; V/V))
  In einen 1‑l-Messkolben wird etwas Acetonitril vorgelegt und 1 ml der 30%igen Ammoniumhydroxid-Lösung hinzugegeben. Anschließend wird der Messkolben bis zur Markierung mit Acetonitril aufgefüllt.

Die Lösungen sind bei 25 °C mindestens sechs Monate stabil.

- Eluent F (Essigsäure (0,5 %))
  In einen 1‑l-Messkolben wird etwas hochreines Wasser vorgelegt und 5 ml Eisessig werden hinzugegeben. Anschließend wird der Messkolben bis zur Markierung mit hochreinem Wasser aufgefüllt.

Die Lösung ist bei 25 °C mindestens sechs Monate stabil.

- Essigsäure (0,5 mol/l)
  In einen 50‑ml-Messkolben wird hochreines Wasser vorgelegt und es werden 1,43 ml des Eisessigs hinzupipettiert. Anschließend wird der Messkolben bis zur Markierung mit hochreinem Wasser aufgefüllt.

Die Lösung ist bei 4 °C mindestens drei Monate stabil.

- Natriumacetat-Lösung (0,5 mol/l)
  3,4 g Natriumacetat‑Trihydrat werden exakt abgewogen und in einen 50‑ml-Messkolben gegeben. Der Messkolben wird anschließend mit hochreinem Wasser bis zur Markierung aufgefüllt.

Die Lösung ist bei 4 °C mindestens drei Monate stabil.

- Natriumacetatpuffer (pH 5,5)
  25 ml der Natriumacetat-Lösung (0,5 mol/l) sowie 13,75 ml der Essigsäure (0,5 mol/l) werden in einem Messkolben miteinander gemischt.

Die Natriumacetatpufferlösung ist bei 4 °C mindestens drei Monate stabil.

- *β*‑Glucuronidase-Lösung
  1 ml der *β*‑Glucuronidase und 27 ml Natriumacetatpuffer werden in einem Messkolben miteinander gemischt.

Die *β*‑Glucuronidase-Lösung ist bei 4 °C mindestens sechs Monate stabil.

### 4.4 Interne Standards (ISTDs)

- Penconazol‑D_7_-Stammlösung (100 mg/l)
  Etwa 1 mg Penconazol‑D_7_ wird exakt in einen 10 ml-Messkolben eingewogen und in Methanol gelöst. Anschließend wird der Messkolben mit Methanol bis zur Markierung aufgefüllt.
- ISTD‑Dotierlösung (10 mg/l)
  1 ml des Tebuconazol‑D_6_-Standards und 1 ml der Penconazol‑D_7_-Stammlösung werden in einen 10‑ml-Messkolben pipettiert. Der Kolben wird anschließend bis zur Markierung mit Methanol aufgefüllt.

Die Penconazol-D_7_-Stammlösung und die ISTD-Dotierlösung werden bei −20 °C im Dunkeln gelagert. Unter diesen Bedingungen sind die Lösungen mindestens sechs Monate stabil.

### 4.5 Kalibrierstandards

- Stammlösungen I (1000 mg/l)
  Jeweils 10 mg TEB‑OH, TEB‑COOH, PEN‑OH sowie PEN‑COOH werden in jeweils einen 10‑ml-Messkolben eingewogen und in ein wenig Methanol gelöst. Die Messkolben werden anschließend mit Methanol bis zur Markierung aufgefüllt.
- Stammlösungen II (100 mg/l)
  Jede Stammlösung I wird einzeln verdünnt, indem 1 ml der jeweiligen Lösung in einen 10‑ml-Messkolben pipettiert wird. Die Messkolben werden anschließend mit Methanol bis zur Markierung aufgefüllt.
- Dotierlösung I (10 mg/l TEB‑OH; 4 mg/l TEB‑COOH; 10 mg/l PEN‑OH; 10 mg/l PEN‑COOH)
  1000 μl der TEB‑OH-Stammlösung II, 400 μl der TEB‑COOH-Stammlösung II, 1000 μl der PEN‑OH-Stammlösung II und 1000 μl der PEN‑COOH-Stammlösung II werden in einen 10‑ml-Messkolben pipettiert. Der Messkolben wird anschließend bis zur Markierung mit Methanol aufgefüllt.
- Dotierlösung II (1 mg/l TEB‑OH; 0,4 mg/l TEB‑COOH; 1 mg/l PEN‑OH; 1 mg/l PEN‑COOH)
  1 ml der Dotierlösung I wird in einen 10‑ml-Messkolben pipettiert. Der Messkolben wird anschließend bis zur Markierung mit Methanol aufgefüllt.
- Dotierlösung III (1 mg/l TEB‑OH; 0,4 mg/l TEB‑COOH; 0,1 mg/l PEN‑OH; 0,1 mg/l PEN‑COOH)
  100 μl der TEB‑OH-Stammlösung II, 40 μl der TEB‑COOH-Stammlösung II, 10 μl der PEN‑OH-Stammlösung II und 10 μl der PEN‑COOH-Stammlösung II werden in einen 10‑ml-Messkolben pipettiert. Der Messkolben wird anschließend bis zur Markierung mit Methanol aufgefüllt.

Die Stamm- und Dotierlösungen werden bei −20 °C im Dunkeln gelagert und sind unter diesen Bedingungen mindestens sechs Monate stabil.

Die Kalibrierstandards werden in gepooltem Urin angesetzt. Für den Poolurin werden Spoturinproben von Personen ohne berufliche Exposition gegen Tebuconazol und Penconazol in geeigneten Behältern gesammelt, gemischt und bei −20 °C bis zum Ansetzen der Kalibrierstandards und Kontrollmaterialien gelagert.

Entsprechend dem in Tabelle 5 gegebenen Pipettierschema werden Kalibrierstandards in Konzentrationsbereichen von 10–500 μg TEB‑OH pro Liter Urin, 4–200 μg TEB‑COOH pro Liter Urin, 1–500 μg PEN‑OH pro Liter Urin sowie 1–500 μg PEN‑COOH pro Liter Urin durch Dotierung des Poolurins angesetzt. Darüber hinaus wird der undotierte Poolurin als Leerwert mitgeführt.

**Tab.5:** Pipettierschema für die Herstellung der Kalibrierstandards für die Bestimmung von TEB‑OH, TEB‑COOH, PEN‑OH und PEN‑COOH in Urin

Kalibrierstandard	Dotierlösung	Dotierlösung [μl]	Poolurin [μl]	Analytkonzentration [μg/l]			
				TEB‑OH	TEB‑COOH	PEN‑OH	PEN‑COOH
0	–	–	1000	–	–	–	–
1	III	10	990	10	4	1	1
2	II	25	975	25	10	25	25
3	II	50	950	50	20	50	50
4	I	10	990	100	40	100	100
5	I	20	980	200	80	200	200
6	I	50	950	500	200	500	500

## 5 Probenahme und Probenaufbereitung

### 5.1 Probenahme

Die Urinproben werden in Urinbechern gesammelt und bis zur Analyse bei −20 °C im Dunkeln gelagert.

### 5.2 Probenaufbereitung

Vor der Analyse werden die Urinproben bei Raumtemperatur aufgetaut und gründlich gemischt. Für die Probenaufarbeitung wird 1 ml der Urinprobe in ein 2‑ml-Bördelrandgläschen pipettiert und mit 5 μl der ISTD‑Dotierlösung sowie 100 μl der *β*‑Glucuronidase-Lösung versetzt. Das Gläschen wird verschlossen und die Probe auf einem Vortexmischer gründlich gemischt. Anschließend wird die Probe bei 37 °C über Nacht inkubiert (16 h) und nach Abkühlen auf Raumtemperatur direkt in das LC‑MS/MS-System injiziert.

## 6 Instrumentelle Arbeitsbedingungen

Die analytische Bestimmung erfolgt an einer Gerätekonfiguration bestehend aus einer TurboFlow-LC-Anlage und einem Tandem-Massenspektrometer (TurboFlow‑LC‑MS/MS). Die Turbulent-Flow-Chromatographie ist eine relativ neue Technik, mit der biologisches Probenmaterial online extrahiert und aufgereinigt wird. Dadurch wird die Probenaufbereitung – ähnlich wie bei einer Online-SPE – deutlich vereinfacht und verkürzt. Die entsprechenden TurboFlow^TM^-Säulen können in LC-Geräten aller Hersteller verwendet werden. Es muss jedoch darauf hingewiesen werden, dass die einzigen auf dem Markt erhältlichen Geräte, die die Vorteile der TurboFlow^TM^-Säulen voll ausschöpfen können, die von Thermo Fisher Scientific vertriebenen Transcend TLX-Systeme sind.

Die hier beschriebene TurboFlow-Methode verwendet eine TurboFlow^TM^-Cyclone-Säule auf Styrol/Divinylbenzol-Copolymer-Basis, die für unpolare Analyten in komplexen Matrizes geeignet ist (pH‑Bereich von 1 bis 13).

### 6.1 Flüssigkeitschromatographie

Das TurboFlow-System verwendet eine quaternäre Pumpe (Beladungspumpe), um die Probe auf die TurboFlow^TM^-Säule aufzugeben und auch zur Extraktion/Reinigung der Probe. Eine zweite, binäre Pumpe (Elutionspumpe) dient dazu, den Probenextrakt auf einer herkömmlichen analytischen Säule aufzutrennen.

**Table:** 

Anreicherungssäule (TurboFlow^TM^-Säule):	TFLC Cyclone Column, 50 × 0,5 mm (auf Styrol/Divinylbenzol-Copolymer-Basis)
Analytische Säule:	Hypersil Gold PFP, 3 μm, 50 × 2,1 mm
Mobile Phasen der quaternären Pumpe P1:	Eluent A: Hochreines Wasser
	Eluent B: Acetonitril
	Eluent C: Acetonitril : 2‑Propanol : Aceton (45 : 45 : 10; V/V/V)
	Eluent D: Acetonitril : Ammoniumhydroxid (99,97 : 0,03; V/V)
Mobile Phasen der binären Pumpe P2:	Eluent E: Methanol
	Eluent F: 0,5 %ige Essigsäure
Flussrate der quaternären Pumpe P1:	siehe Tabelle 6
Flussrate der binären Pumpe P2:	0,7 ml/min
Säulentemperatur:	Raumtemperatur
Injektionsvolumen:	50 μl
Gradientenprogramm:	siehe Tabelle 6
Schaltprogramm der Ventile:	siehe Tabelle 7

**Tab.6:** Gradientenprogramm für die Bestimmung von TEB‑OH, TEB‑COOH, PEN‑OH und PEN‑COOH in Urin

Zeit [min]	Probenaufreinigung mittels TurboFlow^TM^-Säule – quaternäre Pumpe P1							Chromatographische Trennung – binäre Pumpe P2		
	Flussrate [ml/min]	Eluent A [%]	Eluent B [%]	Eluent C [%]	Eluent D [%]	T-Stück	Schleife	Gradient	Eluent E [%]	Eluent F [%]
0,0	1,0	100	0	0	0	–	out	Stufe	0	100
1,0	0,15	100	0	0	0	–	out	Stufe	0	100
1,08	0,15	100	0	0	0	T	in	Stufe	0	100
3,75	1,0	0	0	100	0	–	in	linear	70	30
4,92	1,0	0	0	100	0	–	in	Stufe	70	30
12,0	1,0	0	0	0	100	–	in	Stufe	100	0
13,0	1,0	30	70	0	0	–	in	linear	0	100
14,0	1,0	100	0	0	0	–	out	Stufe	0	100
16,0	1,0	100	0	0	0	–	out	Stufe	0	100

Der für dieses Verfahren verwendete TurboFlow-Chromatographiemodus wird als „Fokusmodus“ bezeichnet, da die Analyten vor der chromatographischen Trennung im Kopf der analytischen Säule fokussiert werden. Der Fokusmodus besteht aus sechs Schritten: Probenaufgabe, Waschschritt (Probe), Überführen der Probe, Waschschritt (System), Befüllen der Elutionsschleife und Rekonditionierung.

**Abb. 2:**
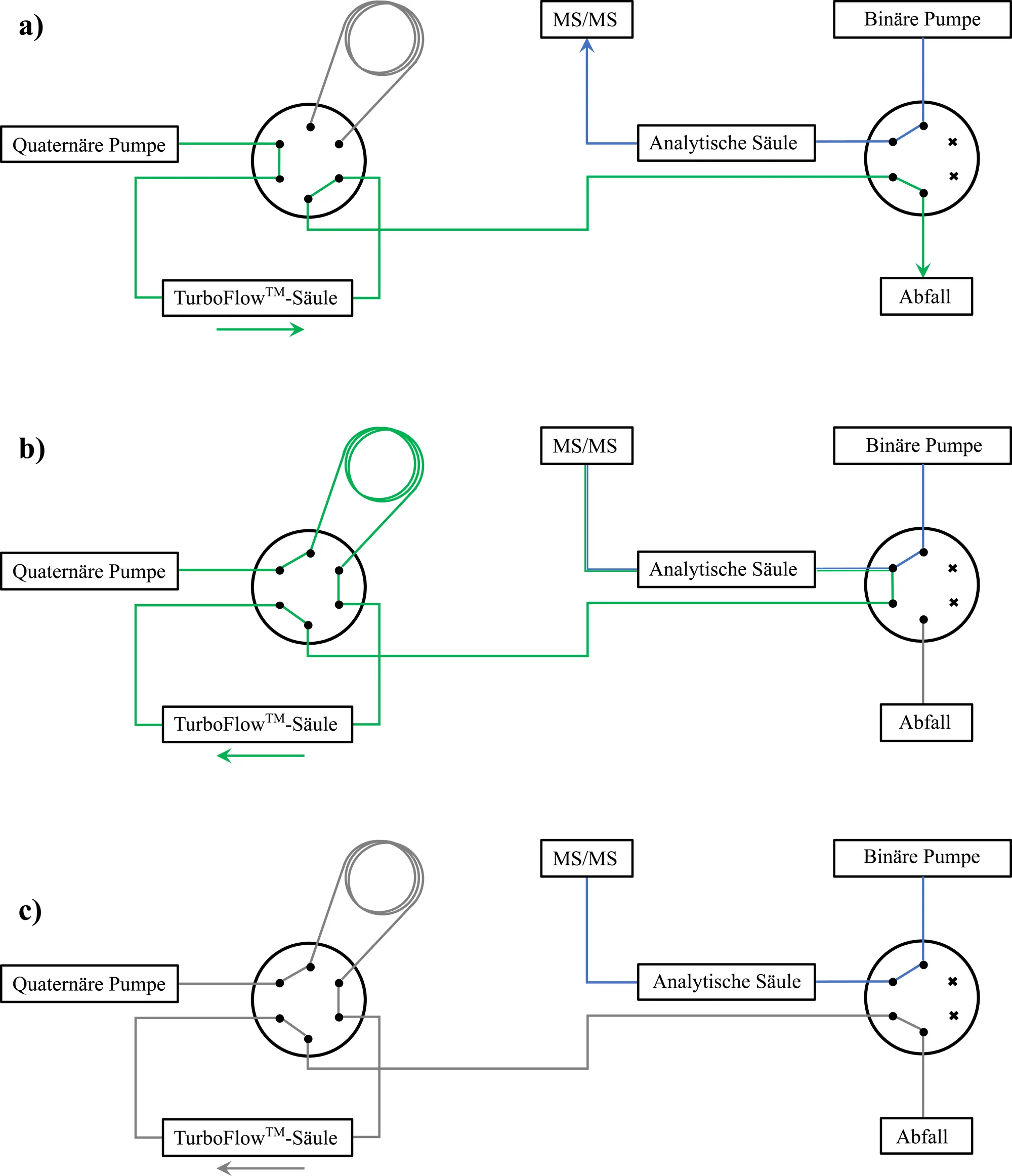
Flussdiagramm der LC-TurboFlow-Anlage: a) Probenaufgabe und Aufreinigung, b) Übertragung auf die analytische Säule im Backflush-Modus, c) chromatographische Trennung gefolgt von Äquilibrierung

Bei der Probenaufgabe werden die Proben mit hoher Flussrate in die TurboFlow^TM^-Säule injiziert, so dass Makromoleküle der Matrix, Salze und ionische Verbindungen, die nicht auf der Säule zurückgehalten werden, in den Abfall fließen. Nach dem Aufgeben wird die Probe durch die mobile Phase von eventuellen Verunreinigungen befreit. Während dieser beiden Schritte wird die mit dem Detektor verbundene analytische Säule mit dem Eluenten F über die binäre Pumpe gespült (Abbildung 2, a).

Für den nachfolgenden Transferschritt werden die Ventile A und B umgeschaltet. Das Lösungsmittelgemisch aus der Schleife wird durch die mobile Phase der quaternären Pumpe im Backflush-Modus in die TurboFlow^TM^-Säule gedrückt. Die Analyten werden eluiert und auf die analytische Säule übertragen (Abbildung 2, b). Bei diesem Schritt wird das Lösungsmittelgemisch der Schleife vor dem Erreichen der analytischen Säule durch die mobile Phase der binären Pumpe (über ein T-Stück im B-Ventil) verdünnt, um die Lösemittelstärke zu verringern und die Zielanalyten im Säulenkopf der analytischen Säule zu fokussieren.

Nach Abschluss des Probentransfers wird das B-Ventil umgeschaltet und der Aufgabe- und der Elutionsfluss werden wieder getrennt. Während die Analyten auf der analytischen Säule aufgetrennt und zum Detektor eluiert werden, werden Schleife und TurboFlow^TM^-Säule gespült, um Verschleppungen zu vermeiden (Abbildung 2, c). Anschließend wird die Schleife erneut mit dem Eluenten für den Transferschritt gefüllt, der für die folgende Probe verwendet werden soll (Abbildung 2, c). Im letzten Schritt wird die Schleife isoliert und die Säulen werden wieder auf die Bedingungen des Probenaufgabeschritts äquilibriert (Abbildung 2, a).

**Tab.7:** Schaltprogramm der Ventile

Zeit [min]	Ventilschaltung	Beschreibung
0,00–1,08	A	Anreicherung und Aufreinigung der Analyten auf der TurboFlow^TM^‑Säule
1,08–3,75	A, B	Überführung der Analyten auf die analytische Säule im Backflush-Modus
3,75–16,0	B	Chromatographische Trennung der Analyten, Spülen und Äquilibrieren der Anreicherungssäule

### 6.2 Tandem-Massenspektrometrie

**Table:** 

Ionisierungsmodus:	Positive Elektrospray-Wärmeionisierung (*heated electrospray ionisation*, HESI+)
Quellentemperatur:	199 °C
Desolvatisierungstemperatur:	211 °C
Detektionsmodus:	*Multiple Reaction Monitoring* (MRM)
Hüllgasdruck:	25 (willkürliche Skala)
Hilfsgasdruck:	15 (willkürliche Skala)
Sprüh-Spannung:	4500 V
Tube-Lens-Offset:	104 V

Sämtliche Ionenquelleneinstellungen und MRM‑Parameter sind gerätespezifisch und müssen vom Anwender der Methode individuell eingestellt werden. Die in diesem Abschnitt genannten Parameter dienen daher nur als Anhaltspunkt. Gegebenenfalls müssen weitere Parameter entsprechend der Gerätespezifikationen des Herstellers optimiert werden.

## 7 Analytische Bestimmung

50 μl der nach Abschnitt 5 aufgearbeiteten Probe werden in das LC‑MS/MS-System injiziert. Die Identifizierung der Analyten erfolgt anhand ihrer Retentionszeiten und spezifischen Massenübergänge. Die Retentionszeiten der Analyten und ISTDs sowie die verwendeten Ionenspuren sind in Tabelle 8 gezeigt. Neben den Kalibrierstandards werden mehrere Leerwertproben (hochreines Wasser anstelle einer Urinprobe) und zwei Qualitätskontrollproben in jeder Analysenserie mitgeführt (siehe Abschnitt 10).

**Tab.8:** Retentionszeiten, Ionenübergänge und Kollisionsenergie für die Bestimmung von TEB‑OH, TEB‑COOH, PEN‑OH und PEN‑COOH in Urin

Analyt bzw. ISTD	Retentionszeit [min]	Ionenspur (*m/z*)		Kollisionsenergie [eV]
		Q1	Q3	
TEB‑OH	5,25	324	70^a)^	21
		324	151	30
TEB‑COOH	5,20	338	70^a)^	27
		338	163	28
Tebuconazol‑D_6_	5,38	314	72^a)^	22
		314	125	25
		314	154	30
PEN‑OH	5,07	300	70^a)^	15
		300	159	27
PEN‑COOH	5,01	314	70^a)^	13
		314	159	29
Penconazol‑D_7_	5,50	291	70^a)^	15
		291	159	33

a) Quantifier

**Abb. 3:**
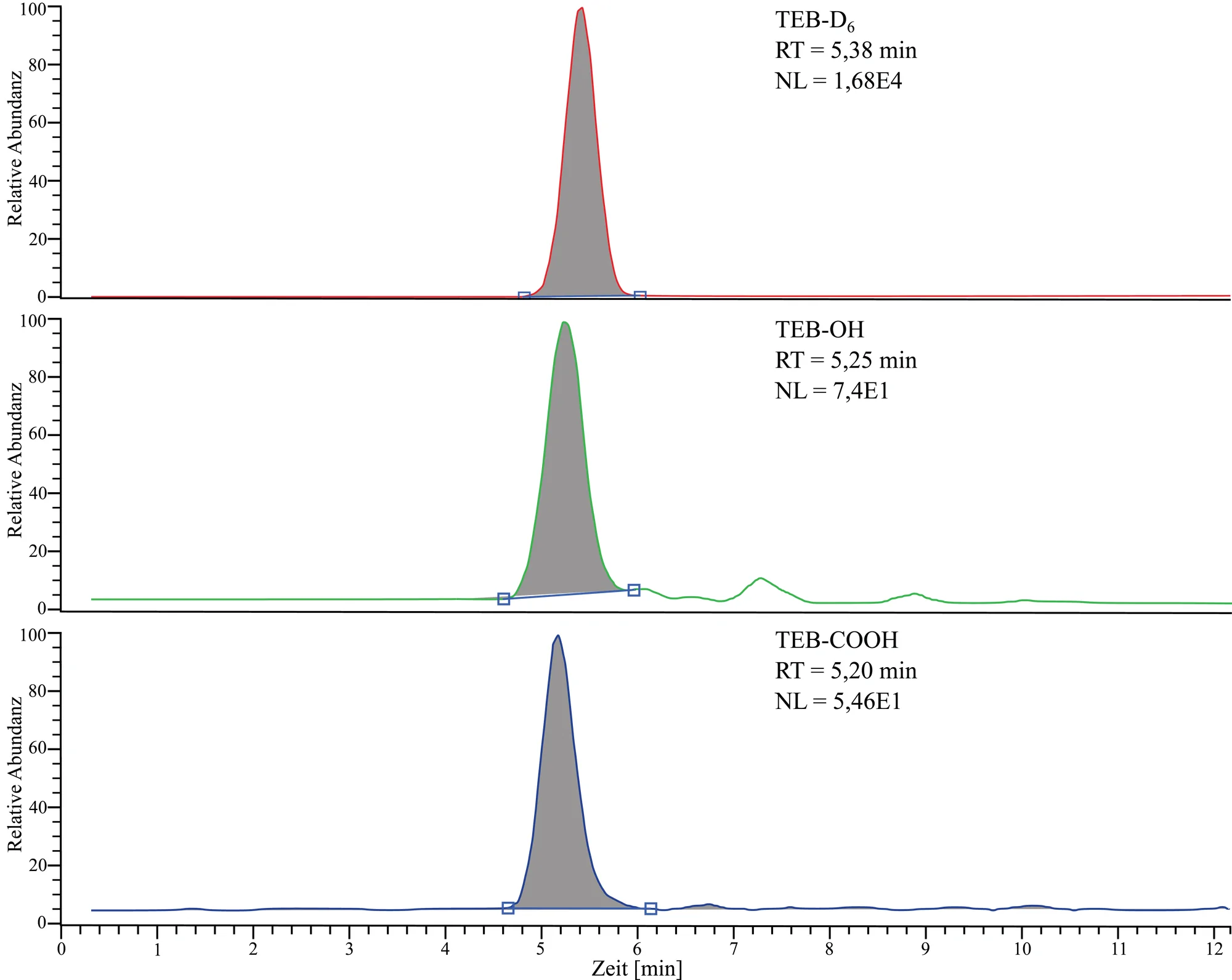
Chromatogramme einer mit TEB-OH und TEB-COOH in Hoehe der Bestimmungsgrenze dotierten Urinprobe unter Angabe von Retentionszeit (RT) und Normalisierungslevel (NL)

Die in Tabelle 8 angegebenen Retentionszeiten dienen nur als Anhaltspunkt und können je nach verwendeter LC‑Gerätekonfiguration variieren. Der Anwender hat sich selbst von der Trennleistung der verwendeten analytischen Säule und dem daraus resultierenden Retentionsverhalten der Analyten zu überzeugen. Abbildungen 3 und 4 zeigen exemplarisch Chromatogramme von einer mit den Analyten in Höhe der Bestimmungsgrenze dotierten Urinprobe.

**Abb. 4:**
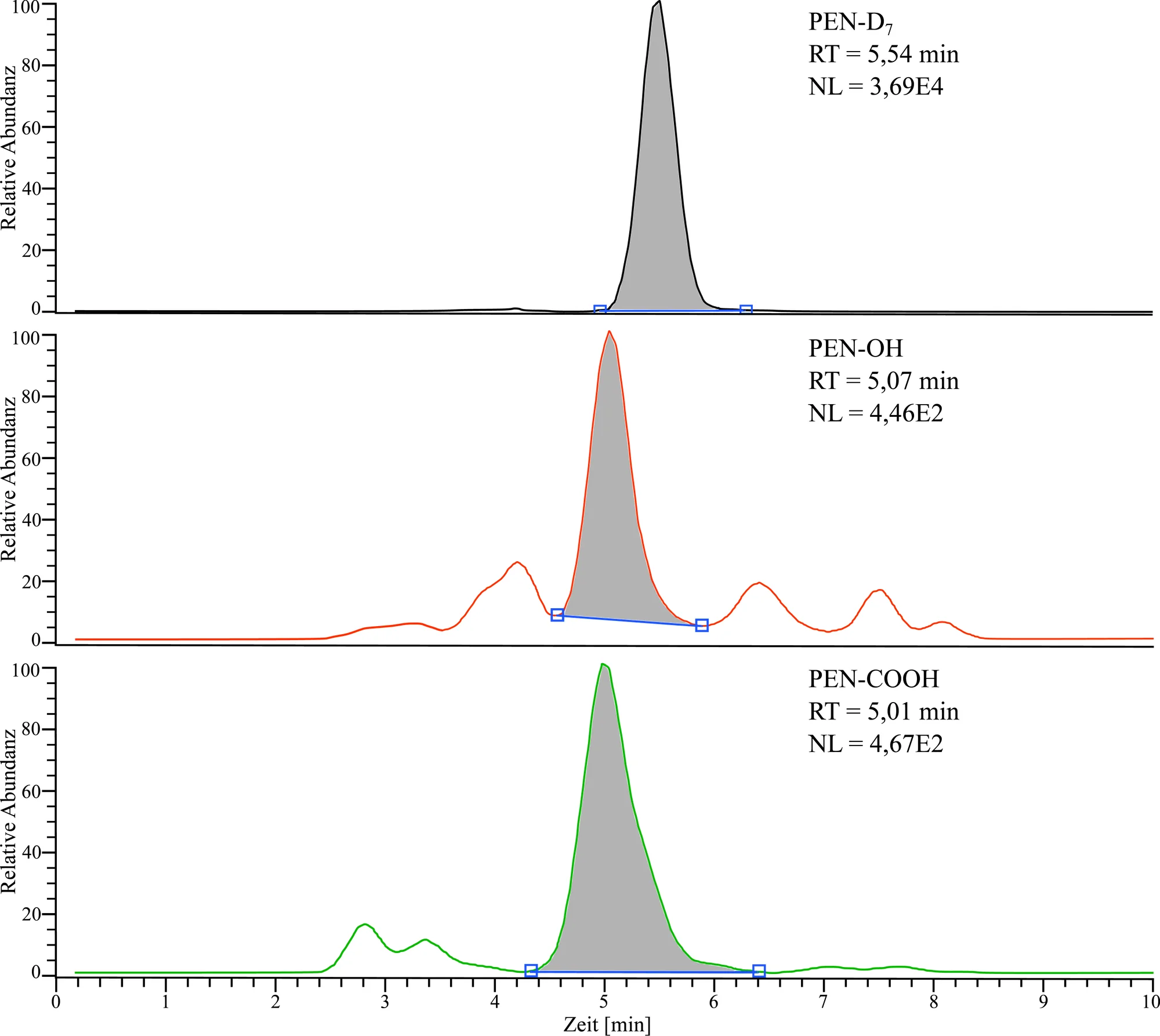
Chromatogramme einer mit PEN-OH und PEN-COOH in Hoehe der Bestimmungsgrenze dotierten Urinprobe unter Angabe von Retentionszeit (RT) und Normalisierungslevel (NL)

## 8 Kalibrierung

**Abb. 5:**
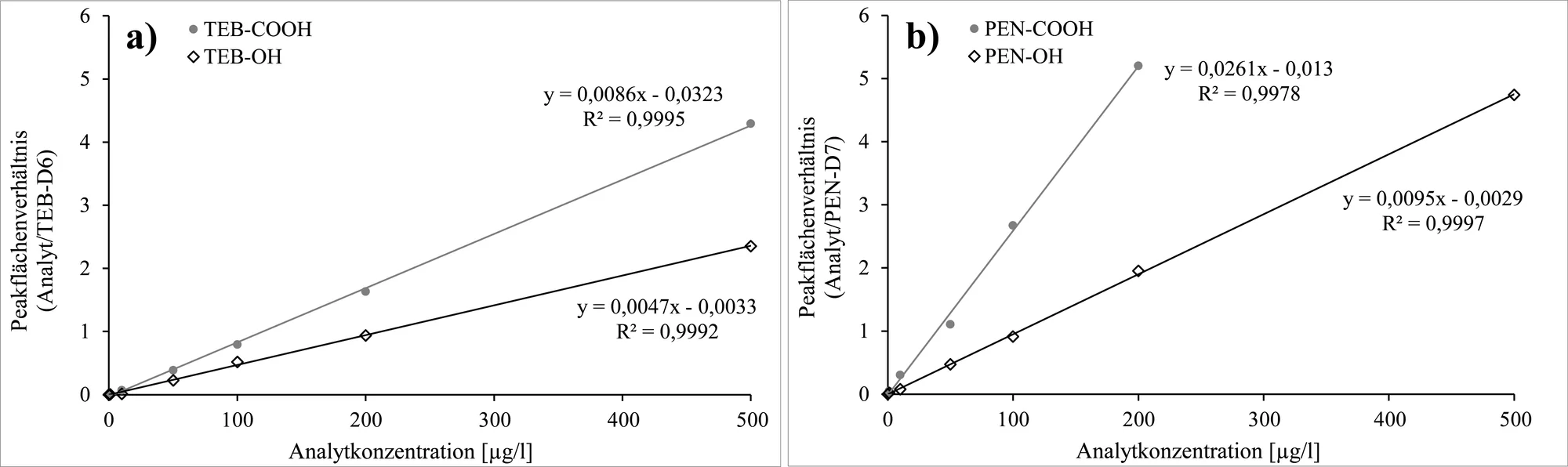
Kalibriergeraden für die Bestimmung von TEB‑OH und TEB-COOH (a) sowie von PEN-OH und PEN-COOH (b) in Urin

Die nach Abschnitt 4.5 hergestellten Kalibrierstandards werden analog zu den Proben aufgearbeitet (siehe Abschnitt 5) und mittels LC‑MS/MS analysiert (siehe Abschnitt 6). Kalibriergeraden werden erstellt, indem die Peakflächenverhältnisse von Analyten und zugehörigen ISTDs gegen die jeweilige Konzentration des Kalibrierstandards aufgetragen werden. Unter den beschriebenen analytischen Bedingungen verlaufen die Kalibriergeraden im Konzentrationsbereich von 10 bis 500 μg/l für TEB‑OH, von 4 bis 200 μg/l für TEB‑COOH, von 1 bis 500 μg/l für PEN‑OH sowie von 1 bis 500 μg/l für PEN‑COOH linear. In Abbildung 5 sind exemplarische Kalibriergeraden für die Bestimmung der Tebuconazol- und Penconazolmetaboliten in Urin dargestellt.

Im Anschluss an die Kalibrierstandards wird bei jeder Analysenserie ein Reagenzienleerwert gemessen, um eine mögliche Verschleppung der Analyten zu überwachen (siehe auch Abschnitt 11.4).

## 9 Berechnung der Analysenergebnisse

Die Berechnung der Analytkonzentrationen in den jeweiligen Urinproben erfolgt mithilfe der zur Analysenserie gehörenden Kalibrierfunktionen (Abschnitt 8). Die Peakfläche des Analyten wird durch die Peakfläche des zugehörigen ISTDs geteilt und das so erhaltene Verhältnis in die Kalibrierfunktion eingesetzt. Man erhält die jeweilige Analytkonzentration in μg/l.

## 10 Standardisierung der Messergebnisse und Qualitätssicherung

Die Qualitätssicherung der Analysenergebnisse erfolgte gemäß den Richtlinien der US-amerikanische Behörde für Lebensmittel- und Arzneimittelsicherheit (*U.S. Food and Drug Administration*, FDA) (FDA 2018).

Zur Präzisionskontrolle werden in jeder Analysenserie mindestens zwei Qualitätskontrollproben mit bekannten Analytkonzentrationen mitgeführt. Da käufliches Material nicht zur Verfügung steht, muss das Qualitätskontrollmaterial selbst hergestellt werden. Dazu wird Urin von Personen ohne berufliche Exposition gegen Tebuconazol oder Penconazol verwendet und mit Standardlösungen der Analyten dotiert, sodass die Analytgehalte im bewertungsrelevanten Konzentrationsbereich liegen.

Von den Entwicklern der Methode wurden folgende Analytkonzentrationen für die Qualitätskontrollmaterialien verwendet: 20 μg TEB‑OH/l, 8 μg TEB‑COOH/l, 5 μg PEN‑OH/l sowie 5 μg PEN‑COOH/l für Q_low_; 150 μg TEB‑OH/l, 60 μg TEB‑COOH/l, 150 μg PEN‑OH/l sowie 150 μg PEN‑COOH/l für Q_high_.

Nach dem Q_high_ sollte ein Reagenzienleerwert analysiert werden, um mögliche Verschleppungseffekte zu erkennen (siehe auch Abschnitt 11.4).

## 11 Beurteilung des Verfahrens

Die Zuverlässigkeit des Verfahrens wurde durch eine umfassende Validierung sowie durch Nachstellung und Prüfung der Methode in einem zweiten, unabhängigen Labor bestätigt. In dem Prüflabor wurde ein UPLC-System von Waters GmbH (Eschborn) in Kombination mit einer TurboFlow^TM^-Säule von Thermo Fisher Scientific GmbH (Dreieich) verwendet. Die instrumentellen Arbeitsbedingungen bei der Prüfung der Methode sind im Anhang dargestellt.

### 11.1 Präzision

Die Präzisionsdaten wurden unter Verwendung der Qualitätskontrollmaterialien ermittelt, indem diese an sieben Tagen fünffach parallel aufgearbeitet und analysiert wurden. In Tabelle 9 ist der für jeden Analyten über die sieben Messtage bestimmte Bereich der Präzision in der Serie angegeben.

**Tab.9:** Präzision in der Serie für die Bestimmung von TEB‑OH, TEB‑COOH, PEN‑OH und PEN‑COOH in Urin (n = 5)

Analyt	Dotierte Konzentration [μg/l]	Standardabweichung (rel.) *s_w_* [%]	Streubereich *u* [%]
TEB‑OH	20	0,1–1,7	0,28–4,72
	150	0,1–1,4	0,28–3,89
TEB‑COOH	8	0,4–3,1	1,11–8,61
	60	0,1–0,9	0,28–2,50
PEN‑OH	5	3,3–4,2	9,16–11,7
	150	2,1–3,5	5,83–9,72
PEN‑COOH	5	3,4–7,5	9,44–20,8
	150	2,1–3,4	5,83–9,44

Zur Ermittlung der Präzision von Tag zu Tag wurde die relative Standardabweichung über sieben Messtage aus den jeweiligen Mittelwerten berechnet. Die so erhaltenen Präzisionsdaten sind in Tabelle 10 angegeben.

**Tab.10:** Präzision von Tag zu Tag für die Bestimmung von TEB‑OH, TEB‑COOH, PEN‑OH und PEN‑COOH in Urin (n = 7)

Analyt	Dotierte Konzentration [μg/l]	Standardabweichung (rel.)* s_w_* [%]	Streubereich *u* [%]
TEB‑OH	20	1,0	2,45
	150	0,8	1,96
TEB‑COOH	8	1,6	3,92
	60	1,4	3,43
PEN‑OH	5	10,7	26,2
	150	4,1	10,0
PEN‑COOH	5	8,7	21,3
	150	7,4	18,1

### 11.2 Richtigkeit

Basierend auf den Daten zur Präzision in der Serie wurden die relativen Wiederfindungen der einzelnen Analyten bestimmt. Die errechneten Daten sind in Tabelle 11 dargestellt.

**Tab.11:** Mittlere relative Wiederfindungen für die Bestimmung von TEB‑OH, TEB‑COOH, PEN‑OH und PEN‑COOH in Urin (n = 5)

Analyt	Dotierte Konzentration [μg/l]	Mittlere Wiederfindung (rel.) *r* [%]	Bereich [%]
TEB‑OH	20	100	99–101
	150	100	99–100
TEB‑COOH	8	100	98–102
	60	101	99–103
PEN‑OH	5	100	91–107
	150	105	103–106
PEN‑COOH	5	106	100–106
	150	107	102–107

### 11.3 Matrixeffekt

Um den Effekt verschiedener Urinmatrices auf die Analysenergebnisse zu ermitteln, wurden sechs Individualurine mit jeweils 20 μg bzw. 150 μg TEB‑OH/l, 8 μg bzw. 60 μg TEB‑COOH/l, 5 μg bzw. 150 μg PEN‑OH/l sowie 5 μg bzw. 150 μg PEN‑COOH/l dotiert, aufgearbeitet und analysiert. Die Ergebnisse sind in Tabelle 12 dargestellt.

**Tab.12:** Präzision in der Serie und relative Wiederfindung für die Bestimmung von TEB‑OH, TEB‑COOH, PEN‑OH und PEN‑COOH in Individualurinproben (n = 6)

Analyt	Dotierte Konzentration [μg/l]	Standardabweichung (rel.) *s_w_* [%]	Mittlere Wiederfindung (rel.) *r* [%]	Bereich [%]
TEB‑OH	20	1,9	101	98–104
	150	1,1	99	97–101
TEB‑COOH	8	1,6	100	97–102
	60	1,2	100	98–101
PEN‑OH	5	1,3	101	99–103
	150	1,2	100	98–102
PEN‑COOH	5	1,4	100	97–102
	150	0,9	99	98–100

### 11.4 Nachweis- und Bestimmungsgrenze

Die Nachweisgrenzen für die Bestimmung der Tebuconazol- und Penconazolmetaboliten wurden aus dem dreifachen Signal/Rausch-Verhältnis ermittelt. Die Bestimmungsgrenzen (BG) der Analyten wurden über die folgende Gleichung bestimmt:

BG = (5 × SE*_q_* + *q*) ÷ *m*

wobei SE*_q_* der Standardfehler des Achsenabschnittes *q* ist und *m* die Steigung der Regressionsgeraden (nach Miller und Miller 2005).

Die für die Analyten berechneten Nachweis- und Bestimmungsgrenzen sind in Tabelle 13 aufgeführt.

**Tab.13:** Nachweis- und Bestimmungsgrenzen für die Bestimmung von TEB‑OH, TEB‑COOH, PEN‑OH und PEN‑COOH in Urin

Analyt	Nachweisgrenze [μg/l]	Bestimmungsgrenze [μg/l]
TEB‑OH	0,2	0,3
TEB‑COOH	0,1	0,3
PEN‑OH	0,5	1,0
PEN‑COOH	0,5	1,0

### 11.5 Störeinflüsse

Zur Extraktion der Analyten aus der Matrix wird in dieser Methode eine Online-Probenextraktion mit TurboFlow^TM^-Säule verwendet. Obwohl eine TurboFlow^TM^‑Säule in LC‑Geräten aller Hersteller verwendet werden kann, wird ihr volles Potential nur mit Transcend TLX-Systemen von Thermo Fisher Scientific ausgeschöpft. Ein solches System kam bei der Methodenentwicklung zum Einsatz.

Die Verwendung des TurboFlow-Systems führte im Vergleich zu der ebenfalls getesteten Offline-Aufarbeitung zu deutlich reduzierten Leerwerten und damit zu niedrigeren Nachweisgrenzen.

Nach Injektion von Proben mit hohen Analytkonzentrationen wurde bei der Entwicklung der Methode eine Verschleppung der Analyten beobachtet. Durch Injektion von hochreinem Wasser – z. B. nach den höchsten Kalibrierstandards – konnte diesem Verschleppungseffekt entgegengewirkt werden. Die Methodenprüfer lösten das Verschleppungsproblem, indem sie deutlich geringere Probenvolumina in das von ihnen verwendete UPLC-System einspritzten. So wurden bei der Methodenprüfung nur 5 μl anstelle von 50 μl aufgegeben, ohne dass die Methodensensitivität verringert wurde (siehe Anhang).

Für TEB‑OH, TEB‑COOH, PEN‑OH und PEN‑COOH waren zur Zeit der Methodenentwicklung keine Isotopen-markierten strukturidentischen Verbindungen als ISTDs kommerziell verfügbar. Daher wurde Tebuconazol‑D_6_ als ISTD für TEB‑OH und TEB‑COOH und Penconazol‑D_7_ für PEN‑OH und PEN‑COOH verwendet. Diese Standards ähnelten den Analyten hinsichtlich ihrer chemischen Struktur nur bedingt (siehe Abbildung 1), wiesen aber auf der verwendeten analytischen Säule ähnliche Retentionszeiten wie die Analyten auf (siehe Tabelle 8 sowie Abbildungen 3 und 4).

Die Prüfung der Methode erfolgte an einem UPLC-System von Waters GmbH (Eschborn) in Kombination mit einer TurboFlow^TM^-Säule von Thermo Fisher Scientific GmbH (Dreieich). Hierbei wurde, vor allem bei der Quantifizierung der Penconazol-Metaboliten, ein Matrixeffekt festgestellt, der durch den verwendeten ISTD Penconazol‑D_7_ nur unzureichend kompensiert wurde und die Leistungsfähigkeit der Methode negativ beeinflusste. Aus diesem Grunde wurde für PEN‑OH und PEN‑COOH die Synthese von strukturidentischen deuterierten ISTDs beauftragt (Details siehe Anhang), die zur Standardisierung der Signale für PEN‑OH und PEN‑COOH verwendet wurden. Die Verwendung von PEN‑OH‑D_6_ und PEN‑COOH‑D_4_ als ISTDs und eine Anpassung der Chromatographie (siehe Anhang) führte zu einer deutlich besseren Wiederfindung der Penconazolmetaboliten. Allerdings war die Quantifizierung von PEN‑OH in einigen Proben durch schlecht abgetrennte Matrixkomponenten nach wie vor gestört. In diesen Fällen ermöglichte die Verwendung der Qualifierspur die korrekte Quantifizierung des Analyten.

In Feldstudien wurde sowohl für die Penconazol- als auch für die Tebuconazolmetaboliten eine zum Teil extrem hohe interindividuelle Variabilität der Konjugatanteile festgestellt (Mercadante et al. 2014, 2016). Demzufolge ist bei der Methode auf eine möglichst vollständige Hydrolyse der Konjugate zu achten.

## 12 Diskussion der Methode

Ziel der Methodenentwicklung war die Erarbeitung einer schnellen und robusten Analysenmethode, um die wichtigsten Tebuconazol- und Penconazolmetaboliten in einem Analysenlauf simultan zu bestimmen (Mercadante et al. 2014). Dabei reduzierte die zur Probenaufreinigung eingesetzte TurboFlow-Technik sowohl den Arbeitsaufwand als auch den Lösungsmittelverbrauch.

Durch die Verwendung von Tebuconazol‑D_6_ und Penconazol‑D_7_ als ISTDs wurden analytische Schwankungen in der Regel gut ausgeglichen. Die Robustheit der entwickelten Analysenmethode wurde durch die Analyse dotierter Individualurinproben bestätigt: bei der Quantifizierung der Analyten in verschiedenen Urinmatrices zeigte sich kein Matrixeffekt.

Die bei der Methodenprüfung eingesetzte Bestimmung der Tebuconazol- und Penconazolmetaboliten ohne TurboFlow-Technik erwies sich unter Einsatz strukturidentischer deuterierter ISTDs als gute Alternative für Labore ohne TurboFlow-Gerät. In der Literatur finden sich weitere Beispiele für die Bestimmung ohne TurboFlow-Technik. Bei der von Sams et al. (2016) entwickelten Analysemethode zur Bestimmung von Penconazol und dessen Metaboliten erfolgte ebenfalls nach enzymatischer Hydrolyse mit *β*‑Glucuronidase (*Helix pomatia*) eine Messung mittels LC‑MS/MS. Dabei wurde für PEN‑COOH eine Nachweisgrenze von 0,25 μg/l berichtet. Im Allgemeinen wurden mit den in der Literatur beschriebenen Methoden entweder nur Tebuconazol- oder Penconazolmetaboliten bestimmt, wobei überwiegend LC‑MS/MS zum Einsatz kam. Diese Methoden erreichen meist vergleichbare oder geringfügig niedrigere Nachweis- und Bestimmungsgrenzen (Alcalá et al. 2024; Bustamante et al. 2025; Rodríguez-Zamora et al. 2024; Sams et al. 2016). Šulc et al. (2022) haben eine um Faktor sechs niedrigere Nachweisgrenze für TEB‑OH publiziert. Mit der hier beschriebenen Methode können jedoch erstmals die beiden Hauptmetaboliten sowohl von Tebuconazol als auch von Penconazol simultan erfasst werden.

**Verwendete Messgeräte**

Methodenentwicklung: LC‑MS/MS-Anlage bestehend aus einem TurboFlow-System mit zwei Gradientpumpen, einem Eluenten-Entgaser und einem Autosampler (Thermo Fisher Scientific S.p.A., Rodano, Italien) sowie einem Tandem-Massenspektrometer (TSQ Quantum Access, Thermo Fisher Scientific S.p.A., Rodano, Italien)

Methodenprüfung: ACQUITY UPLC H‑Class System (Waters GmbH, Eschborn) bestehend aus einer quaternären Pumpe (ACQ H‑Class QSM Plus), einer binären Pumpe (UPLC Binary SOL MGR), einem Autosampler (ACQ H‑Class FTN H‑Plus) sowie einem Säulenmanager (ACQUITY UPLC CM A) mit Triple-Quadrupol-Massenspektrometer (Xevo TQ XS, Waters GmbH, Eschborn)

## Figures and Tables

**Abb. 6:**
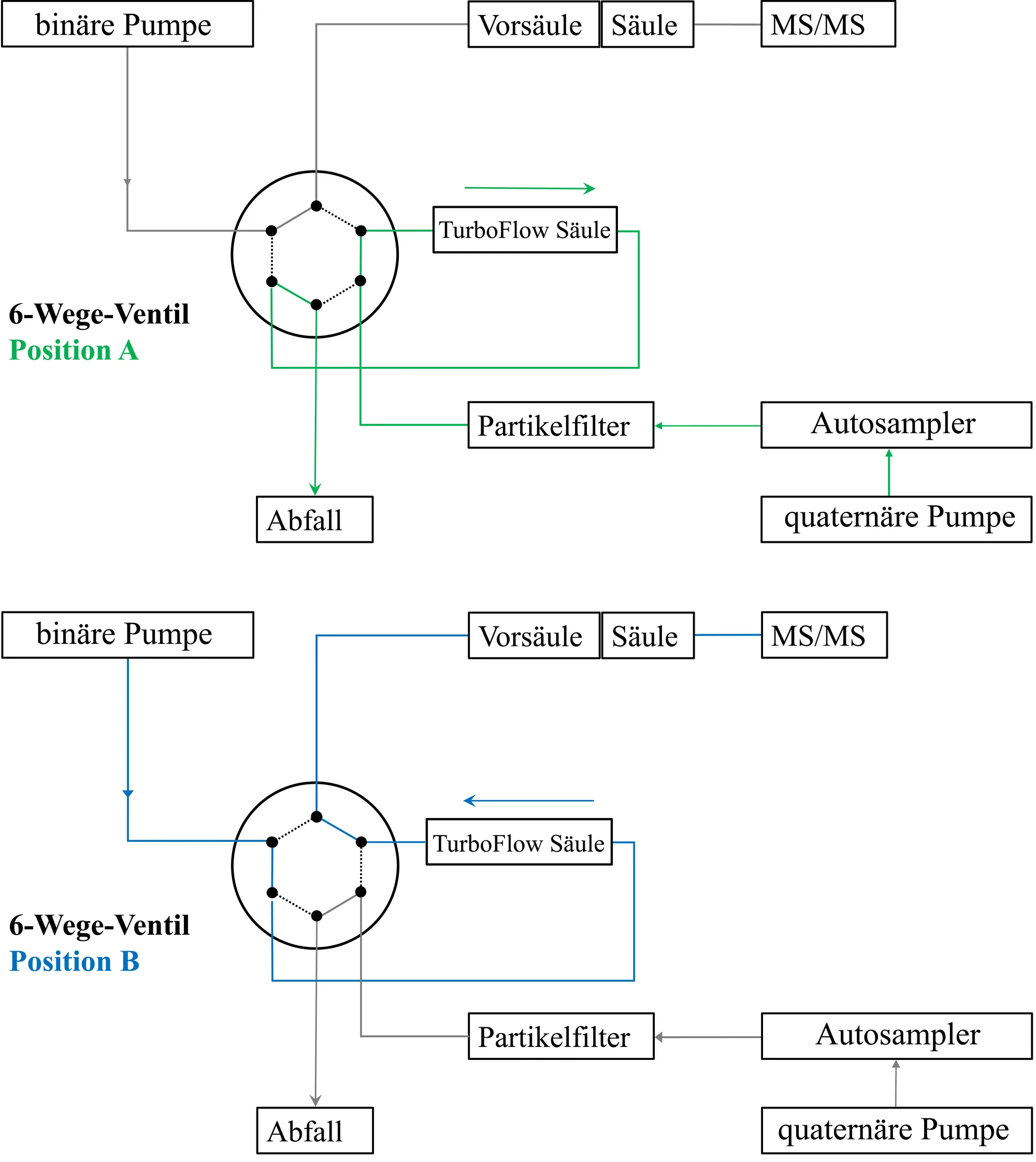
Skizze der LC‑MS/MS-Anlage und Illustration der Schaltpositionen A (grün) und B (blau) des 6‑Wege-Ventils (Kuhlmann et al. 2025)

**Table:** 

Anreicherungssäule:	TFLC Cyclone, 50 × 0,5 mm
Vorsäule:	Hypersil Vorsäule, 3 μm, 10 mm × 2,1 mm
Analytische Säule:	Hypersil Gold PFP, 3 μm, 50 × 2,1 mm
Mobile Phasen der quaternären Pumpe P1:	Eluent A: 0,5 % Essigsäure in Wasser Eluent B: Methanol Eluent C: Acetonitril : Isopropanol (60 : 40; V/V) Eluent D: 0,03 % Ammoniumhydroxid in Acetonitril
Mobile Phasen der binären Pumpe P2:	Eluent E: 0,5 % Essigsäure in Wasser Eluent F: Methanol
Flussrate der quaternären Pumpe P1:	siehe Tabelle 14
Flussrate der binären Pumpe P2:	siehe Tabelle 15
Säulentemperatur:	Raumtemperatur
Injektionsvolumen:	5 μl
Gradientenprogramm:	siehe Tabelle 14 und 15
Schaltprogramm der Ventile:	siehe Tabelle 16

**Tab.14:** Gradientenprogramm der quaternären Pumpe P1 (Methodenprüfung)

Zeit [min]	Flussrate [ml/min]	Eluent A [%]	Eluent B [%]	Eluent C [%]	Eluent D [%]
Beginn	1,00	95	5	–	–
1,50	1,00	95	5	–	–
1,58	0,15	95	5	–	–
3,30	0,15	–	–	100	–
3,75	1,00	–	–	100	–
4,92	1,00	–	–	100	–
5,00	1,00	–	–	–	100
6,00	1,00	–	–	–	100
6,10	1,00	30	70	–	–
7,00	1,00	30	70	–	–
7,50	1,00	95	5	–	–
10,0	1,00	95	5	–	–

**Tab.15:** Gradientenprogramm der binären Pumpe P2 (Methodenprüfung)

Zeit [min]	Flussrate [ml/min]	Eluent E [%]	Eluent F [%]
Beginn	0,50	40	60
1,00	0,50	40	60
4,00	0,50	40	60
4,10	0,50	20	80
5,00	0,50	0	100
8,00	0,50	0	100
8,50	0,50	40	60
10,0	0,50	40	60

**Tab.16:** Schaltprogramm der Ventile (Methodenprüfung)

Zeit [min]	Ventilposition	Beschreibung
Initial	A	Anreicherung und Reinigung der Analyten auf der Anreicherungssäule
1,50–3,75	B	Überführung der Analyten von der Anreicherungssäule auf die analytische Säule im Backflush-Modus
3,75–10,0	A	Chromatographische Trennung der Analyten, Spülen und Äquilibrieren der Anreicherungssäule

**Table:** 

Ionisierungsmodus:	Positive Elektrosprayionisierung (ESI+)
Kapillarspannung:	3,5 kV
Quellentemperatur:	150 °C
Desolvatisierungstemperatur:	600 °C
Cone-Gas-Fluss:	150 l/h
Desolvatisierungsgas-Fluss:	1000 l/h
Zerstäubergas:	Stickstoff, 7,00 bar
Kollisionsgas:	Argon, 0,15 ml/min

**Tab.17:** Retentionszeiten, Ionenübergänge und Kollisionsenergie für die Bestimmung von TEB‑OH, TEB‑COOH, PEN‑OH und PEN‑COOH in Urin

Analyt bzw. ISTD	Retentionszeit [min]	Ionenspur (*m/z*)		Cone-Spannung [V]	Kollisionsenergie [eV]
		Q1	Q3		
TEB‑OH	2,74	324	70^a)^	20	18
		324	179	20	22
		324	125	20	40
TEB‑COOH	2,68	338	125^a)^	20	42
		338	70	20	18
		338	163	20	22
Tebuconazol‑D_6_	3,42	314	72^a)^	20	20
		314	125	20	36
		314	154	20	26
PEN‑OH	2,25	300	70^a)^	20	14
		300	143	20	32
		300	159	20	28
PEN‑COOH	2,24	314	159^a)^	18	36
		314	185	18	24
		314	70	18	16
PEN-OH‑D_6_	2,25	306	70^a)^	38	14
		306	148	38	36
		306	160	38	34
PEN-COOH‑D_4_	2,24	320	70^a)^	18	20
		320	151	18	36
		320	159	18	38

a) Quantifier

**Abb. 7:**
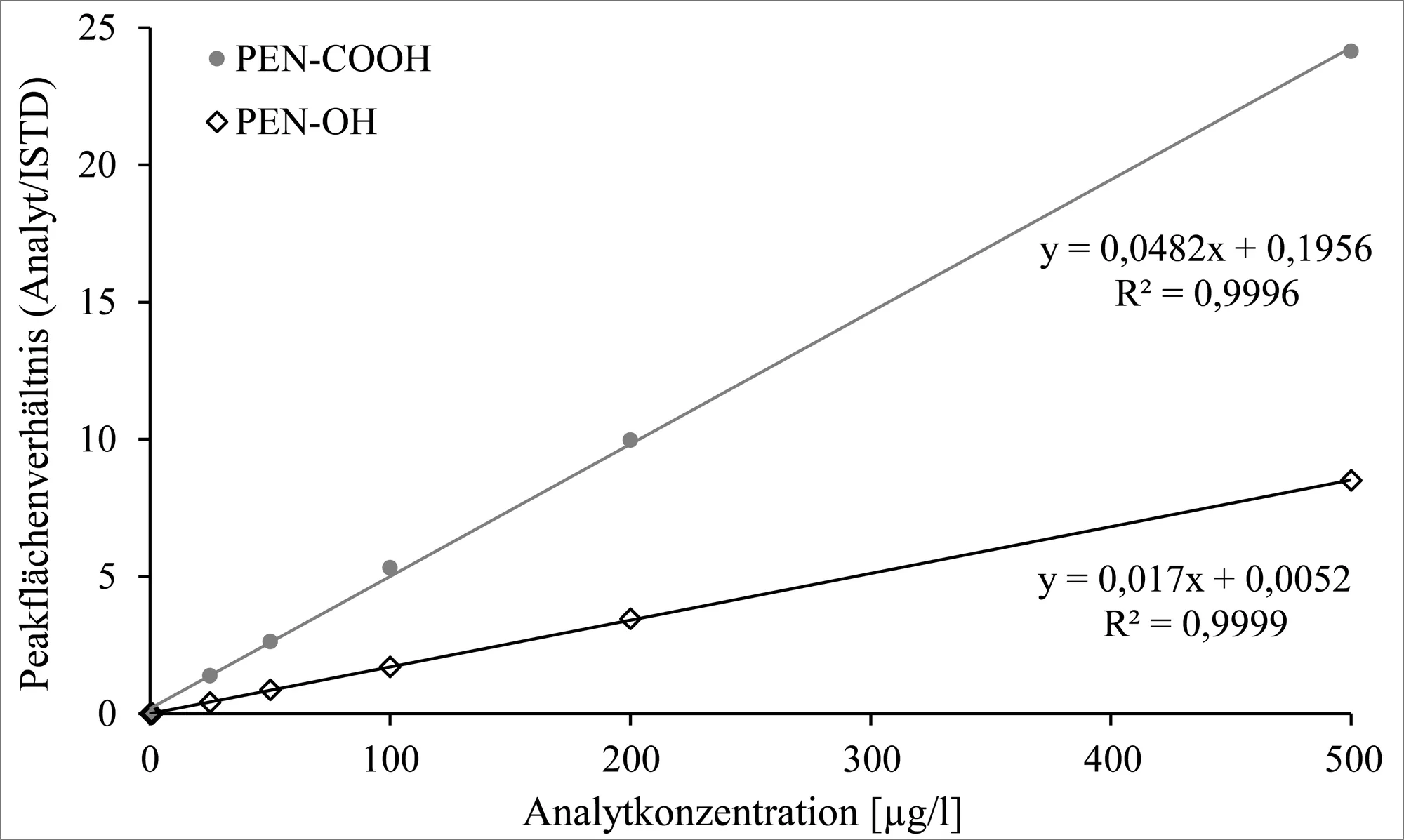
Kalibriergeraden für die Bestimmung von PEN-OH und PEN-COOH in Urin unter Verwendung strukturidentischer deuterierter ISTDs

**Tab.18:** Nachweis- und Bestimmungsgrenzen für die Bestimmung von TEB‑OH, TEB‑COOH, PEN‑OH und PEN‑COOH in Urin

Analyt	Nachweisgrenze [μg/l]	Bestimmungsgrenze [μg/l]
TEB‑OH	0,07	0,21
TEB‑COOH	0,08	0,26
PEN‑OH	0,05	0,16
PEN‑COOH	0,09	0,27

## Anhang

Bei der Methodenprüfung wurde eine UPLC-Anlage von Waters GmbH (Eschborn) in Kombination mit einer TurboFlow^TM^-Säule von Thermo Fisher Scientific GmbH (Dreieich) eingesetzt. Infolge dessen mussten chromatographische Parameter für die Anreicherung, den Analyttransfer und die Analytentrennung angepasst werden. Die zugehörigen instrumentellen Arbeitsbedingungen sind im Folgenden detailliert aufgeführt, um Methodennutzern ohne Transcend TLX-System die Anwendung der Methode zu erleichtern.

### Geräte, Chemikalien und Lösungen

#### Geräte

- LC‑Anlage (ACQUITY UPLC H-Class System) bestehend aus einer quaternären Pumpe (ACQ H-Class QSM Plus), einer binären Pumpe (UPLC Binary SOL MGR), einem Autosampler (ACQ H-Class FTN H-Plus) sowie einem Säulenmanager (ACQUITY UPLC CM A) (z. B. Waters GmbH, Eschborn)
- Triple-Quadrupol-Massenspektrometer (z. B. Xevo TQ XS, Waters GmbH, Eschborn)
- In-Line-Filter (z. B. Nr. 205000343, ACQUITY Column In-Line-Filter Waters Critical Clean^TM^, Waters GmbH, Eschborn)
- Anreicherungssäule (z. B. Nr. CH‑953288, TurboFlow^TM^-Cyclone-Säule, Länge: 50 mm, Innendurchmesser: 0,5 mm, Thermo Fisher Scientific GmbH, Dreieich)
- Vorsäule (z. B. Nr. 25403‑012101, Hypersil Vorsäule, 3 μm, 10 mm × 2,1 mm, Thermo Fisher Scientific GmbH, Dreieich)
- Analytische Säule (z. B. Nr. 25403‑052130, Hypersil Gold PFP, 3 μm, 50 × 2,1 mm, Thermo Fisher Scientific GmbH, Dreieich)

#### Chemikalien

- *β*‑Glucuronidase/Arylsulfatase aus *Helix pomatia* (≥ 100 000 U/ml Glucuronidaseaktivität) (z. B. Nr. BGALA‑RO, Roche Diagnostics Deutschland GmbH, Mannheim)
- 4-(2,4-Dichlorphenyl)-5-(1*H*-1,2,4-triazol-1-yl)pentan-1,1,2,2,3,3-D_6_-1-ol (PEN‑OH‑D_6_) (z. B. Auftragssynthese, Max-Planck-Institut für Multidisziplinäre Naturwissenschaften, Abteilung für Synthesechemie, Göttingen)
- 4-(2,4-Dichlorphenyl)-5-(1*H*-1,2,4-triazol-1-yl)pentansäure-2,2,3,3-D_4_ (PEN‑COOH‑D_4_) (z. B. Auftragssynthese, Max-Planck-Institut für Multidisziplinäre Naturwissenschaften, Abteilung für Synthesechemie, Göttingen)

#### Lösungen

- Eluent A (Wasser : Methanol : Essigsäure (94,5 ∶ 5 ∶ 0,5; V/V/V))
  In einen 500‑ml-Messkolben werden 250 ml hochreines Wasser vorgelegt. Anschließend werden 25 ml Methanol und 2,5 ml Eisessig zugegeben und der Kolben wird anschließend bis zur Markierung mit hochreinem Wasser aufgefüllt.

Die Lösung ist bei Raumtemperatur mindestens drei Monate stabil.

- Eluent C (Acetonitril : Isopropanol (60 : 40; V/V))
  In einen 500‑ml-Messkolben werden 300 ml Acetonitril vorgelegt. Der Kolben wird anschließend bis zur Markierung mit Isopropanol aufgefüllt.
- Eluent D (0,03 % Ammoniumhydroxid in Acetonitril)
  In einen 1‑l-Messkolben werden 500 ml Acetonitril vorgelegt und 1 ml der 30%igen Ammoniumhydroxid-Lösung wird hinzugegeben. Anschließend wird der Messkolben bis zur Markierung mit Acetonitril aufgefüllt.
- Eluent E (0,5 % Essigsäure in Wasser)
  In einen 500‑ml-Messkolben werden 250 ml hochreines Wasser vorgelegt und 2,5 ml Eisessig hinzugegeben. Anschließend wird der Messkolben bis zur Markierung mit hochreinem Wasser aufgefüllt.

Die Lösung ist bei Raumtemperatur mindestens drei Monate stabil.

#### Interne Standards (ISTDs)

- PEN‑OH‑D_6_-Stammlösung (1000 mg/l)
  Etwa 5 mg PEN‑OH‑D_6 _werden in einen 5‑ml‑Messkolben exakt eingewogen und in Acetonitril gelöst. Anschließend wird der Messkolben bis zur Markierung mit Acetonitril aufgefüllt.
- PEN‑COOH‑D_4_-Stammlösung (1000 mg/l)
  Etwa 5 mg PEN‑COOH‑D_4 _werden in einen 5‑ml‑Messkolben exakt eingewogen und in Acetonitril gelöst. Anschließend wird der Messkolben bis zur Markierung mit Acetonitril aufgefüllt.
- ISTD‑Dotierlösung (5 mg/l)
  Zu einem Milliliter der Tebuconazol‑D_6_/Penconazol‑D_7_-Dotierlösung (10 mg/l, siehe Abschnitt 4.4) werden 10 μl der PEN‑OH‑D_6_-Stammlösung, 10 μl der PEN‑COOH‑D_4_-Stammlösung und 980 μl Methanol pipettiert.

Die PEN‑OH‑D_6_- und die PEN‑COOH‑D_4_-Stammlösungen sowie die ISTD-Dotierlösung werden bei −20 °C im Dunkeln gelagert. Unter diesen Bedingungen sind die Lösungen mindestens sechs Monate stabil.

### Probenahme und Probenaufbereitung

#### Probenaufbereitung

Die Probenaufbereitung entspricht dem in Abschnitt 5.2 beschriebenen Vorgehen. Um eine präzisere Pipettierbarkeit zu gewährleisten, wurden den Proben 10 μl der geringer konzentrierten ISTD-Dotierlösung (5 mg/l) zugesetzt.

### Instrumentelle Arbeitsbedingungen

Die Methode wurde an einer ACQUITY UPLC H‑Class Anlage (Waters GmbH, Eschborn) geprüft, bestehend aus einer quaternären Pumpe (ACQ H‑Class QSM Plus), einer binären Pumpe (UPLC Binary SOL MGR), einem Autosampler (ACQ H‑Class FTN H‑Plus) sowie einem Säulenmanager (ACQUITY UPLC CM A) mit Triple-Quadrupol-Massenspektrometer (Xevo TQ XS, Waters GmbH, Eschborn).

Die quaternäre Pumpe (Beladungspumpe) wurde verwendet, um die Probe auf die TurboFlow ^TM^-Säule aufzugeben und anzureichern. Die binäre Pumpe (Elutionspumpe) diente dazu, die Probenextrakte auf einer herkömmlichen analytischen Säule aufzutrennen (Abbildung 6).

#### Flüssigkeitschromatographie

Es empfiehlt sich, vor der Anreicherungssäule einen Partikelfilter einzufügen, um Säulen und Anlagenteile zu ­schützen. Alle anderen Parameter sind nach Herstellerangaben zu optimieren.

### Analytische Bestimmung

5 μl der aufgearbeiteten Probe werden in das LC‑MS/MS-System injiziert. Die Identifizierung der Analyten erfolgt anhand ihrer Retentionszeiten und spezifischen Massenübergänge. Die Retentionszeiten der Analyten und ISTDs sowie die verwendeten Ionenspuren sind in Tabelle 17 aufgeführt.

### Kalibrierung und Berechnung der Analysenergebnisse

Für die Tebuconazolmetaboliten erfolgt die Kalibrierung und Berechnung der Analysenergebnisse wie unter Abschnitt 8 und 9 beschrieben. Für die Penconazolmetaboliten werden die Kalibriergeraden erstellt, indem die Peakflächenverhältnisse von PEN‑OH bzw. PEN‑COOH und den dazugehörenden ISTDs (PEN‑OH‑D_6_ bzw. PEN‑COOH‑D_4_) gegen die jeweilige Konzentration des Kalibrierstandards aufgetragen werden. Die linearen Arbeitsbereiche für die Bestimmung der Tebuconazol- und Penconazolmetaboliten entsprachen den von den Methodenentwicklern angegebenen Konzentrationsbereichen. Beispielhafte Kalibriergeraden für die Bestimmung von PEN‑OH und PEN‑COOH unter Verwendung strukturidentischer deuterierter ISTDs sind in Abbildung 7 gezeigt.

### Qualitätssicherung

Zur Sicherung der Qualität der Analysenergebnisse wurde gemäß den Richtlinien der Bundesärztekammer und den Angaben in dem vor der Kommission veröffentlichten allgemeinen Kapitel verfahren (Bader et al. 2010; Bundesärztekammer 2023).

### Beurteilung des Verfahrens

Die Methodenprüfer haben mit dem von ihnen verwendeten UPLC-MS/MS-System die gesamte Methode umfassend validiert. Dabei entsprachen sowohl die Präzisionsdaten als auch die Analytwiederfindungen den von den Methodenentwicklern gefundenen guten Zuverlässigkeitskriterien. Die Untersuchung von dotierten Individualurinen durch die Methodenprüfer ergab ebenfalls präzise und richtige Ergebnisse ohne Hinweis auf einen Matrixeffekt.

Bei der Methodenprüfung wurden die Nachweis- und Bestimmungsgrenzen mit der Kalibriergeradenmethode bestimmt. Die äquidistanten zehn-Punkt-Kalibriergeraden überspannten einen Konzentrationsbereich von 0,1–1,0 μg/l. Die in Tabelle 18 aufgeführten Nachweis- und Bestimmungsgrenzen für die Tebuconazolmetaboliten waren mit den von den Methodenentwicklern angegebenen Werten vergleichbar, während sich für die Penconazolmetaboliten bessere Nachweis- und Bestimmungsgrenzen ergaben.

Zusammenfassend kann festgehalten werden, dass die angepasste UPLC‑Methode ebenfalls die präzise und richtige Quantifizierung der wichtigsten Tebuconazol- und Penconazol-Metaboliten ermöglicht.
